# Genome-wide survey of sucrose non-fermenting 1-related protein kinase 2 in Rosaceae and expression analysis of *PbrSnRK2* in response to ABA stress

**DOI:** 10.1186/s12864-020-07201-w

**Published:** 2020-11-10

**Authors:** Guodong Chen, Jizhong Wang, Xin Qiao, Cong Jin, Weike Duan, Xiaochuan Sun, Juyou Wu

**Affiliations:** 1 College of Life Science and Food Engineering, Huaiyin Institute of Technology, Huai’an, 223003 China; 2 Center of Pear Engineering Technology Research, State Key Laboratory of Crop Genetics and Germplasm Enhancement, College of Horticulture, Nanjing Agricultural University, Nanjing, 210095 China

**Keywords:** SnRK2, Pear, Expression analysis, ABA, Abiotic stress

## Abstract

**Background:**

The members of the sucrose non-fermenting 1-related protein kinase 2 (SnRK2) family are specific serine/threonine protein kinases in plants that play important roles in stress signal transduction and adaptation. Because of their positive regulatory roles in response to adverse conditions, the genes encoding thes proteins are considered potential candidates for breeding of plants for disease resistance and genetic improvement. However, there is far less information about this kinase family, and the function of these genes has not been explored in Rosaceae.

**Results:**

A genome-wide survey and analysis of the genes encoding members of the SnRK2 family were performed in pear (*Pyrus bretschneideri*) and seven other Rosaceae species. A total of 71 *SnRK2* genes were identified from the eight Rosaceae species and classified into three subgroups based on phylogenetic analysis and structural characteristics. Purifying selection played a crucial role in the evolution of *SnRK2* genes, and whole-genome duplication and dispersed duplication were the primary forces underlying the characteristics of the *SnRK2* gene family in Rosaceae. Transcriptome data and qRT-PCR assay results revealed that the distribution of PbrSnRK2s was very extensive, including across the roots, leaves, pollen, styles, and flowers, although most of them were mainly expressed in leaves. In addition, under stress conditions, the transcript levels of some of the genes were upregulated in leaves in response to ABA treatment.

**Conclusions:**

This study provides useful information and a theoretical introduction for the study of the evolution, expression, and functions of the *SnRK2* gene family in plants.

**Supplementary Information:**

The online version contains supplementary material available at 10.1186/s12864-020-07201-w.

## Background

During growth and agricultural production, plants are frequently affected by various biotic and abiotic stresses such as waterlogging, salinity, and cold and drought stresses. Because of their immovable nature and inability to choose suitable environmental conditions, plants must change their characteristics to adapt to adverse environments. To cope with these stresses, plants have established a network of defense-related metabolic mechanisms by regulating the production of beneficial substances or the expressions of related genes over the long course of evolution [1]. As important adversity signal regulators, protein kinases and phosphorylation play essential roles in the process of recognizing and transmitting stress signals to different parts of cells [2]. The sucrose non-fermenting 1-related protein kinase 2 (SnRK2) family is a plant-specific serine/threonine (Ser/Thr) family of protein kinases that are particularly involved in adversity stress responses and play important roles in plant stress signal transduction [3–5]. Recent studies have shown that SnRK2s are involved in plant growth and development as well as in responses to stress signals such as osmotic stress, saline stress and ABA signaling [4, 6]. For example, SnRK2.6 and SnRK2.8 serve as regulators of carbohydrate metabolism and drought resistance response in *Arabidopsis thaliana*, respectively [7, 8], whereas SnRK2.4 and SnRK2.10 play essential roles in root growth and architecture under saline conditions in *Arabidopsis* [9]. Furthermore, in other species, overexpression of SnRK2.8 enhances the resistance to various adverse stresses in wheat (*Triticum aestivum*) [10], while ZmSAPK8, OsSAPK8, GhSnRK2.6, and MpSnRK2.10 are related to salt tolerance in maize (*Zea mays*), rice (*Oryza sativa*), cotton (*Gossypium hirsutum*), and apple (*Malus domestica*), respectively [11–14].

Similar to other SnRK family members, SnRK2 proteins possess a typical structure composed of structurally and functionally conserved domains. The amino acid sequence of SnRK2s can be divided into two regions: the highly conserved N-terminal catalytic domain, which is similar to that of SNF1/AMP kinases, and the relatively differentiated C-terminal regulatory domain, which is short [15]. In addition, earlier research found that the N-terminus of SnRK2 is a highly conserved kinase region, which is closely related to its kinase activity, and contains the highly conserved ATP-binding site (DXGXGNFGVAXL) and Ser/Thr kinase activity domain (KICDFGYSKSXXXHGXPK) [16]. It was also found that the C-terminus was composed of two subdomains (Domains I and II), which contain stretches of acidic patches of either glutamic acid or aspartic acid [16]. Domain I is a region shared by all members of the SnRK2 family that is located 20 amino acids away from the catalytic domain and is the necessary structural basis for independent ABA response to osmotic stress [17]. Domain II exists mainly in the members of the third subfamily and is the necessary structural basis for the response to ABA signals [17].

Differences in the functions of *SnRK2* genes have been widely determined. In contrast to other protein kinases, SnRK2s are essential components and positive regulators of the ABA signaling pathway that are specific to plants. Based on the structural characteristics of the SnRK2 C-terminus and their response to ABA signals, SnRK2s were divided into three subfamilies [14]. Among them, members of Group I are rich in glutamic acid at the C-terminus and barely respond to ABA signals. In contrast, members of Group II and Group III are rich in aspartic acid; SnRK2s in Group II do not or weakly respond to ABA signals, whereas SnRK2s in Group III are strongly activated by ABA [4, 18]. Furthermore, the promoter region of SnRK2s usually contains *cis*-acting regulatory elements that are involved in stress responses, such as drought-responsive elements, ABA-responsive elements, and cold-responsive elements [6]. Previous research has shown that the kinase activity of SnRK2 is related to the reversible phosphorylation of key amino acid sites. For example, the kinase activity of NtOSAK is regulated by the phosphorylation of Ser154 and Ser158 within its activated ring in the tobacco (*Nicotiana tabacum*) [19]. Furthermore, there are some differences in the kinase activity of SnRK2s because of their different phosphorylation sites. Regarding the *SnRK2.6* gene in *Arabidopsis*, the phosphorylation sites Ser7, Ser18, Ser29, and Ser43 are mainly related to the regulation of self-phosphorylation, whereas Ser175 and Thr176 are primarily associated with kinase activity [20].

Because of the critical regulatory functions of *SnRK2* genes in plant responses to various adversity stresses and developmental processes, the *SnRK2* gene family has been widely studied in the model plant *Arabidopsis* as well as in non-model plants such as rice, maize, wheat, soybean (*Glycine max*), apple, and rubber (*Hevea brasiliensis*) [6, 15, 18, 21–23]. Compared with other species, the members of the *SnRK2* gene family have not been extensively examined in Rosaceae. Pear (*Pyrus bretschneideri*) is a member of the Rosaceae family and a commercially important crop that is cultivated in temperate regions worldwide. In addition, the genome of the Chinese white pear (*P*. *bretschneideri* Rehd. cv. ‘Dangshansuli’) has been fully sequenced [24]. In recent years, the development of high-throughput sequencing technology has allowed genome sequencing in many organisms for genomic analysis. For example, genome sequences are available for seven other Rosaceae species, i.e., apple, strawberry (*Fragaria vesca*), peach (*Prunus persica*), Chinese plum (*Prunus mume*), black raspberry (*Rubus occidentalis*), cherry (*Prunus avium*), and European pear (*Pyrus communis*), which provides an opportunity to further analyze the *SnRK2* gene family in Rosaceae. Therefore, the present study aimed to (1) annotate the full-length *SnRK2* genes in Chinese white pear and seven additional Rosaceae fruit species; (2) infer their expansion, evolutionary history, and expression patterns; and (3) explore PbrSnRK2s responses to adversity stresses, as elicited by ABA stress, thereby providing valuable information for further investigation of *SnRK2* gene functions in Rosaceae. These results will be useful for revealing the mechanisms of stress resistance in fruit trees and will lay a foundation for further investigations that will use genetic engineering for molecular breeding.

## Results

### Identification and classification of *SnRK2* genes in Rosaceae

**Fig. 1:**
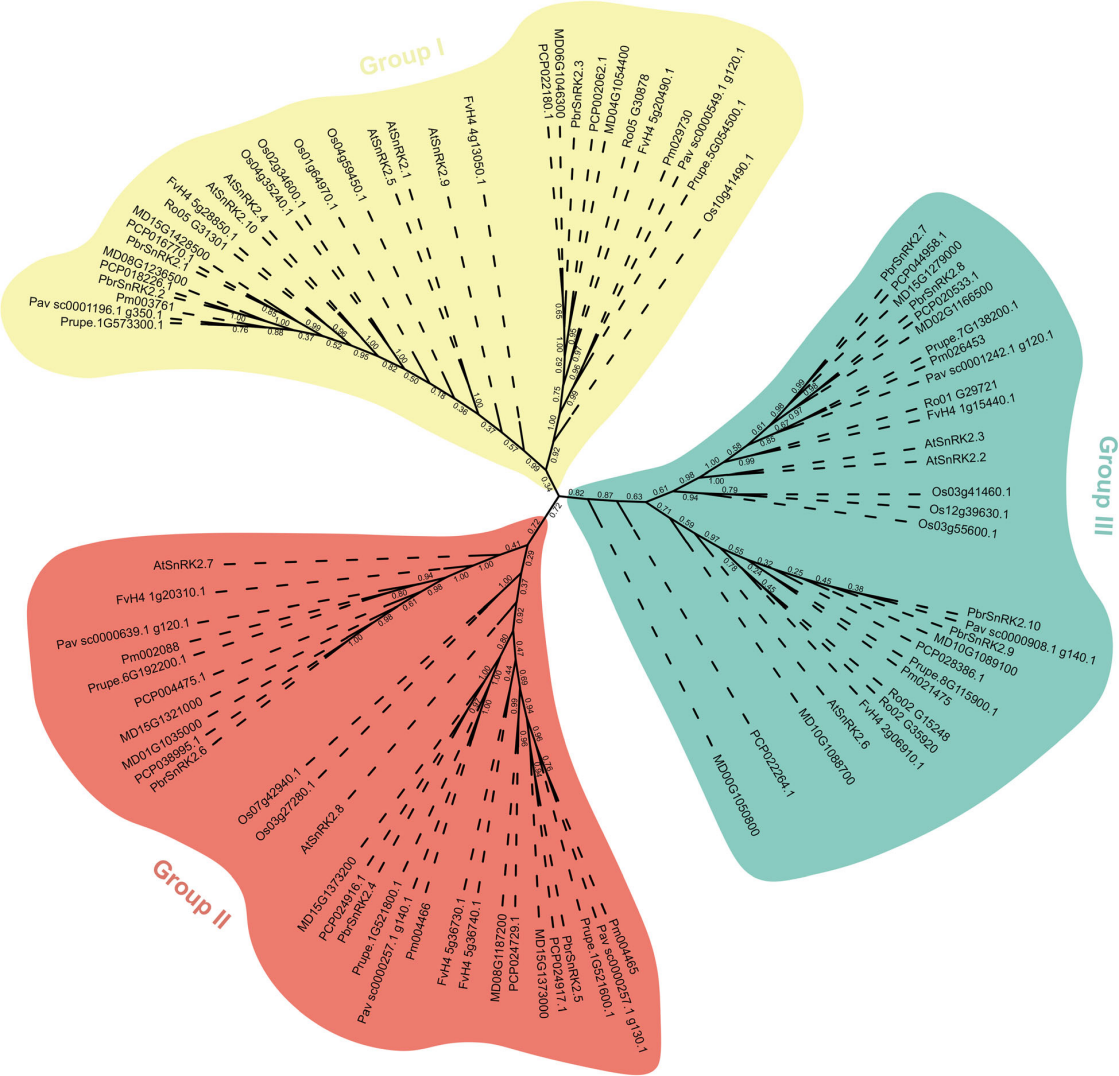
Phylogenetic analysis of SnRK2 proteins from Rosaceae, rice, and *Arabidopsis*. Full-length protein sequences were aligned with the integrated MUSCLE program and the phylogenetic tree was constructed by the maximum likelihood method using MEGA 6.0 and 1000 bootstrap replicates. Proteins clustered into three subgroups. The yellow, red, and green regions indicate the three subfamilies of the SnRK2 proteins

To investigate the *SnRK2* gene family in Rosaceae, the protein sequences of SnRK2s from *Arabidopsis* and rice were used as queries. In addition, a Hidden Markov Model (HMM) search using the *SnRK2* gene domain HMM profile (PF00069.1) was used to screen the Rosaceae genome. A total of 318 candidate *SnRK2* genes were screened using these two strategies. Finally, the online program SMART (http://smart.embl-heidelberg.de/) was used to assess the Ser/Thr protein kinase catalytic domains, followed by the elimination of redundant sequences, incomplete gene sequences, and transcripts of the same gene. Subsequently, 71 nonredundant *SnRK2* genes were identified in the Rosaceae genome. Among them, 10 SnRK2 proteins were identified in Chinese white pear (PbrSnRK2s), 14 in apple (MdSnRK2s), 8 in strawberry (FvSnRK2s), 7 in peach (PpeSnRK2s), 7 in Chinese plum (PmSnRK2s), 5 in black raspberry (RocSnRK2s), 7 in cherry (PavSnRK2s), and 13 in European pear (PcSnRK2s) (Fig. 1).

To classify and investigate the evolutionary relationships among *SnRK2* genes, a phylogenetic tree was constructed using multiple sequence alignment of SnRK2 protein sequences from the eight Rosaceae species, *Arabidopsis*, and rice. The results of this analysis showed that the *SnRK2* gene family was clustered into three well-supported clades (Groups I, II, and III; Fig. 1), which is consistent with the findings of a previous study performed in *Arabidopsis* and rice [15]. Among them, 22 members belonged to Group I, 25 to Group II, and 24 to Group III (Fig. 1).

### Multiple sequence alignment of SnRK2 proteins

**Fig. 2:**
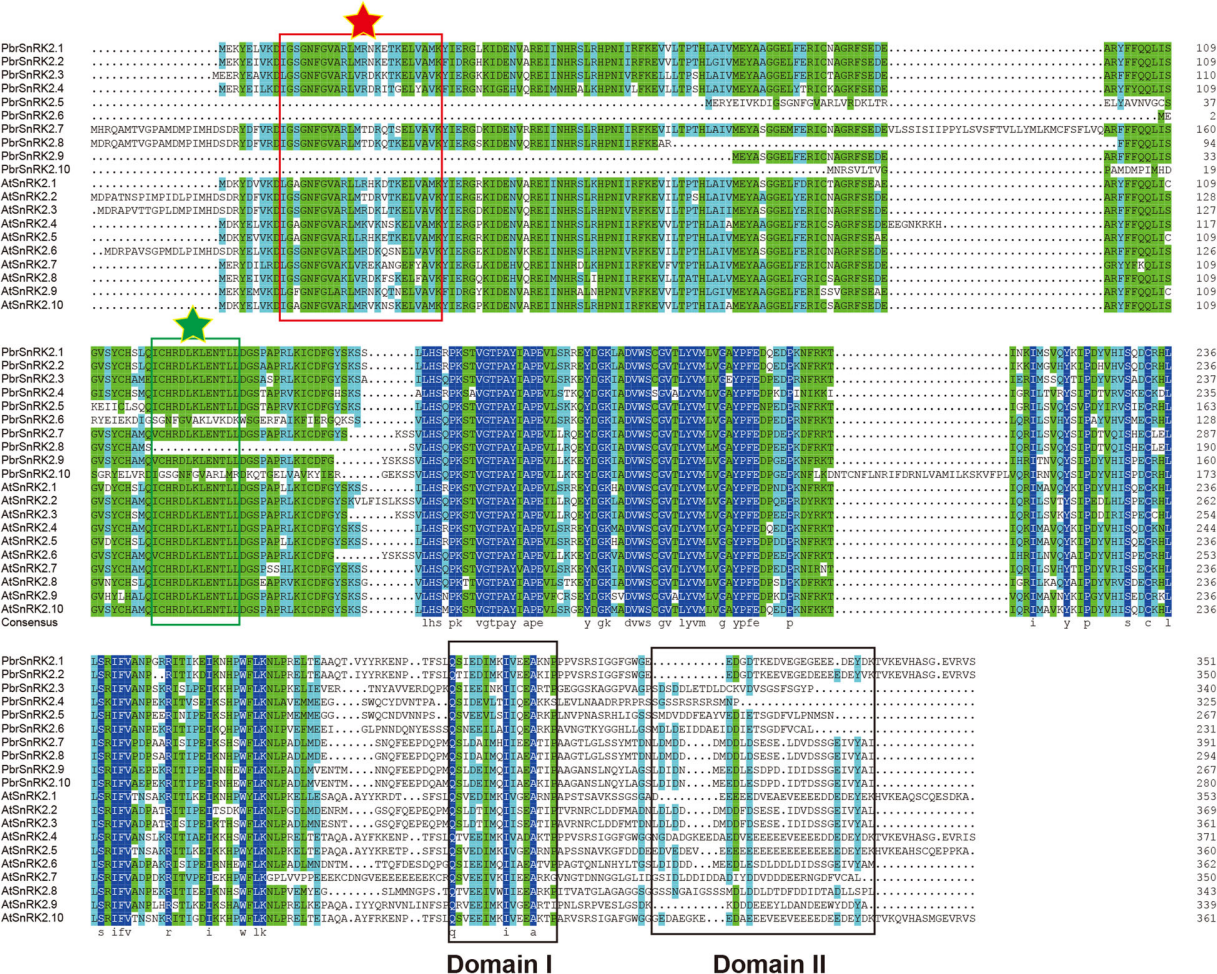
Alignment of the amino acid sequences of PbrSnRK2s and AtSnRK2s. The serine/threonine protein kinase active site signature and the protein kinase ATP-binding region signature are indicated by a green box (a green pentacle) and red box (a red pentacle), respectively. Domain I and Domain II are marked by black boxes at the C-terminus

To gain insights into the structural features of PbrSnRK2 proteins, the amino acid sequences in all members and groups were aligned. Result of the amino acid sequence alignment showed that all members of SnRK2s had 54.87% sequence identity, of which the maximum and minimum percentage of amino acid sequence conserved was 79.78 and 29.33%, respectively (Table S1). Furthermore, the ratio of sequence conservation for subgroup I, II and III was 78.92, 64.5 and 65.17% respectively (Table S1). Previous studies in *Arabidopsis* had identified two conserved kinase regions and two functional subdomains within SnRK2, i.e. ATP-binding site, Ser/Thr kinase activity domain, Domain I and Domain II. To characterize the biological functions of *PbrSnRK2* genes and their conserved domains in pear, multiple sequence alignment of PbrSnRK2 and AtSnRK2 proteins was performed. According to the result, the amino acid sites were very similar in these conserved regions of pear and *Arabidopsis* SnRK2 proteins. Specifically, members of PbrSnRK2 proteins have two conserved kinase domains in the N-terminal regions: an ATP-binding signature containing a lysine residue as an ATP-binding site, expected for the PbrSnRK2.5/2.6/2/9/2/10; and a Ser/Thr protein kinase active site signature, expected for the PbrSnRK2.8 (Fig. 2). In addition, the C-terminal of PbrSnRK2 contains two distinct domains (Domain I and Domain II), which is identical with the SnRK2 members identified in *Arabidopsis* lineages that showed divergent C-terminal domains. Together, the results manifest that *SnRK2* gene family originated before the divergence of Rosaceae and Cruciferae. Additionally, the kinase domain regions are highly conserved during long-term evolution in SnRK2 proteins, which may have gone through purification choices. Furthermore, the conserved regions of several PbrSnRK2 proteins were lost, indicating that pseudogenezation or subfunctionalization might have been occurred during long-term evolution in pear.

### Structural and conserved motifs analysis of SnRK2 genes in pear

**Fig. 3:**
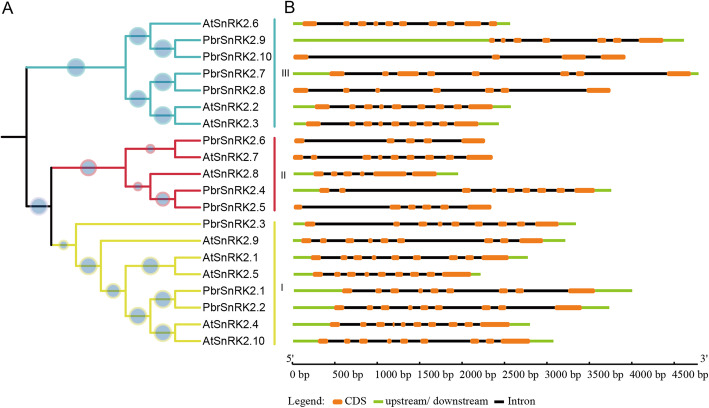
Phylogenetic relationship of the intron/exon structure of *SnRK2* genes from pear and *Arabidopsis thaliana*. **a** A phylogenetic tree constructed with CluxtalX2.0 using the full-length amino acid sequences of *SnRK2* genes from pear and *Arabidopsis*. Bootstrap analysis was performed using 1000 replicates. The species in which SnRK2 proteins were functionally characterized are displayed as icons, and the different colors in the branches represent the different subfamilies. **b** The tangerine boxes, black lines, and light-green boxes in the gene structural diagram represent exons, introns, and UTRs, respectively. Gene models are drawn to scale, as indicated at the bottom

Analysis of the arrangement of introns and exons can provide valuable information regarding evolutions and functions of gene families [25]. To better understand the structural diversity of *PbrSnRK2* genes, an exon/intron analysis was performed by aligning gene sequences with their corresponding coding domains from SnRK2 in pear and *Arabidopsis*. The number of exons identified in the members of the *SnRK2* gene family ranged from 4 to 10 in pear and *Arabidopsis* (Fig. 3). Most members of the individual groups exhibited different number of exons/introns and varying lengths of the coding sequence in pear, which also supported the phylogenetic classification of the *SnRK2* gene family. For example, *SnRK2* genes in subgroup I in pear contained 9 exons. Most members of subgroup II included 5–6 exons, except *PbrSnRK2.4*, which contained 9 exons. In addition, subgroup III contained a number of exons (i.e., 7–8), except *PbrSnRK2.10*, which contained 4 exons (Fig. 2). In addition, the MEME tool was used to further investigate the conserved motifs of SnRK2 proteins. A total of 12 conserved motifs were identified, denominating motif 1–12. The *SnRK2* genes of each subgroup shared similar conserved motifs, nevertheless those in subgroup II were disorganized. Based on the result of motif analysis, motif 1/4/6/8/9/10 were identified to be the basic regions of the SnRK2 domain, as they were detected in each group of the gene family (Fig. S1). For example, it contains 11 of 12 conserved motifs in subgroup I, and only motif 12 is lacked (Fig. S1). Group III contains one specific motifs, i.e. it contains two motifs 12, expected for PbrSnRK2.9 (Fig. S1). In total, the conservation and specificity of the number of exons and motifs in each subgroup support the close evolutionary relationship of *PbrSnRK2* genes. This may result from the replication events in evolution process of the gene family, indicating that these subgroups originated via different evolutionary paths.

### Physicochemical features of the SnRK2 genes in Rosaceae

To further study the functions of the SnRK2 proteins, we performed systematic analysis of the physicochemical properties of the SnRK2 proteins in Rosaceae. We found that the SnRK2 protein sequences ranged from 198 to 891 amino acids, and that most of them contained 220 to 402 amino acids. The isoelectric point of 87.3% of the SnRK2 proteins was acidic, which indicates that SnRK2 proteins from Rosaceae are rich in acidic amino acids. Moreover, the molecular weights of these proteins ranged from 30.09 to 85.3 kDa (Table 1). The negative and positive GRAVY scores of proteins reflect their hydrophobicity and hydrophilicity, respectively [26]. The grand average of the hydropathy scores of all SnRK2 proteins was negative in Rosaceae, which indicates that these proteins are hydrophilic. In addition, we found that the aliphatic index ranged from 80.76 to 94.08 for the SnRK2 proteins from Rosaceae, which indicated that all of them are thermally stable (Table 1).

**Table 1:** Characteristics of the SnRK2 proteins

Gene name	Subfamily	protein length (aa)	Protein Molecular Weight (Da)	PI	GRAVY	Formula	Aliphatic index
Pbr003186.1 (PbrSnRK2.1)	1	351	40,164.76	6.12	−0.493	C_1787_H_2813_N_493_O_534_S_13_	83.56
Pbr026536.1 (PbrSnRK2.2)	1	350	40,114.75	6.15	−0.465	C_1786_H_2811_N_495_O_530_S_13_	84.63
Pbr040276.1 (PbrSnRK2.3)	1	340	38,378.82	6.29	−0.409	C_1705_H_2701_N_469_O_512_S_13_	84.85
Pbr040625.1 (PbrSnRK2.4)	2	325	36,631.27	9.06	−0.287	C_1630_H_2611_N_455_O_475_S_14_	90.89
Pbr040624.1 (PbrSnRK2.5)	2	267	30,085.35	5.45	−0.258	C_1332_H_2101_N_361_O_404_S_14_	90.45
Pbr006341.1 (PbrSnRK2.6)	2	231	26,079.62	5.23	−0.359	C_1167_H_1818_N_310_O_352_S_8_	86.49
Pbr007881.1 (PbrSnRK2.7)	3	391	44,256.56	4.73	−0.112	C_1962_H_3070_N_516_O_598_S_25_	91.74
Pbr025097.1 (PbrSnRK2.8)	3	294	33,287.80	4.57	−0.277	C_1468_H_2298_N_388_O_458_S_18_	86.22
Pbr042784.1 (PbrSnRK2.9)	3	267	30,129.09	4.63	−0.279	C_1339_H_2069_N_349_O_414_S_14_	86.22
Pbr023607.1 (PbrSnRK2.10)	3	280	31,555.06	4.82	−0.249	C_1398_H_2209_N_373_O_427_S_15_	92.25
FvH4_5g20490.1	1	337	38,042.32	5.85	−0.399	C_1687_H_2673_N_467_O_510_S_12_	86.2
FvH4_2g06910.1	3	363	41,228.89	4.88	−0.316	C_1821_H_2858_N_492_O_559_S_20_	88.35
FvH4_5g28850.1	1	327	37,323.51	5.86	−0.487	C_1659_H_2603_N_455_O_497_S_14_	80.76
FvH4_1g20310.1	2	337	38,383.77	5.18	−0.286	C_1710_H_2684_N_456_O_517_S_15_	90.5
FvH4_1g15440.1	3	395	44,944.60	4.78	−0.108	C_2006_H_3107_N_519_O_595_S_29_	86.86
FvH4_5g36730.1	2	891	100,973.94	6.69	−0.278	C_4490_H_7134_N_1238_O_1327_S_40_	90.76
FvH4_4g13050.1	1	364	41,481.68	9.1	−0.429	C_1829_H_2934_N_524_O_540_S_18_	82.17
FvH4_5g36740.1	2	265	30,127.71	8.2	−0.369	C_1335_H_2131_N_373_O_392_S_14_	84.91
MD15G1428500	1	352	40,374.9	6	−0.518	C_1795_H_2819_N_495_O_539_S_13_	81.68
MD08G1236500	1	352	40,335.01	6.15	− 0.487	C_1792_H_2819_N_497_O_533_S_15_	81.93
MD04G1054400	1	340	38,363.68	5.99	−0.436	C_1700_H_2688_N_472_O_513_S_13_	83.74
MD06G1046300	1	341	38,402.85	6.06	−0.396	C_1704_H_2701_N_467_O_514_S_14_	84.34
MD08G1187200	2	338	38,563.34	6.39	−0.283	C_1724_H_2713_N_473_O_499_S_16_	89.35
MD10G1089100	3	374	42,618.45	4.9	−0.282	C_1897_H_2946_N_506_O_573_S_19_	88.61
MD01G1035000	2	339	38,468.89	5.65	−0.307	C_1716_H_2683_N_465_O_510_S_15_	88.58
MD02G1166500	3	359	40,537.12	4.69	−0.275	C_1789_H_2805_N_475_O_554_S_22_	86.1
MD15G1321000	2	339	38,437.74	5.48	−0.354	C_1710_H_2674_N_464_O_514_S_15_	86.58
MD15G1373000	2	339	38,522.06	5.97	−0.247	C_1722_H_2686_N_468_O_504_S_16_	88.5
MD15G1279000	3	359	40,772.25	4.73	−0.291	C_1792_H_2806_N_488_O_555_S_22_	86.91
MD15G1373200	2	323	36,386.03	8.96	−0.26	C_1622_H_2596_N_450_O_471_S_14_	92.35
MD10G1088700	3	383	43,396.23	8.53	−0.15	C_1946_H_3062_N_534_O_549_S_21_	88.8
MD00G1050800	3	220	25,729.73	8.03	−0.079	C_1182_H_1780_N_300_O_320_S_13_	84.14
Pav_sc0001196.1_g350.1	1	317	36,635.72	6.58	−0.587	C_1629_H_2566_N_456_O_484_S_11_	82.4
Pav_sc0001242.1_g120.1	3	295	33,333.86	4.78	−0.336	C_1464_H_2296_N_396_O_455_S_19_	82.64
Pav_sc0000549.1_g120.1	1	262	29,207.29	5.61	−0.437	C_1296_H_2070_N_352_O_398_S_8_	85.61
Pav_sc0000639.1_g120.1	2	278	31,570.93	5.49	−0.267	C_1402_H_2189_N_379_O_423_S_14_	89.82
Pav_sc0000908.1_g140.1	3	285	32,103.42	4.72	−0.354	C_1417_H_2233_N_377_O_444_S_14_	88.91
Pav_sc0000257.1_g140.1	2	445	49,201.30	5.45	−0.042	C_2218_H_3423_N_575_O_649_S_21_	89.8
Pav_sc0000257.1_g130.1	2	226	25,483.15	6.09	−0.39	C_1126_H_1798_N_312_O_341_S_10_	87.08
PCP016770.1	1	351	40,164.76	6.12	−0.493	C_1787_H_2813_N_493_O_534_S_13_	83.56
PCP018226.1	1	375	42,928.18	6.41	−0.338	C_1927_H_3021_N_529_O_557_S_13_	89.39
PCP022180.1	1	341	38,437.84	6.13	−0.403	C_1706_H_2702_N_470_O_514_S_13_	84.63
PCP002062.1	1	340	38,446.75	5.99	−0.44	C_1708_H_2693_N_473_O_513_S_12_	84.32
PCP028386.1	3	362	41,005.69	5.01	−0.293	C_1814_H_2849_N_493_O_552_S_19_	89.67
PCP024729.1	2	338	38,278.91	6.09	−0.259	C_1708_H_2693_N_469_O_500_S_15_	90.77
PCP020533.1	3	359	40,561.10	4.68	−0.27	C_1789_H_2801_N_477_O_554_S_22_	86.38
PCP024917.1	2	762	85,296.85	6.78	−0.248	C_3776_H_6009_N_1053_O_1122_S_37_	88.22
PCP004475.1	2	343	39,053.51	5.4	−0.338	C_1737_H_2711_N_473_O_518_S_17_	87.29
PCP044958.1	3	402	45,383.96	4.67	−0.076	C_2014_H_3160_N_526_O_613_S_26_	94.08
PCP024916.1	2	323	36,388.00	8.96	−0.275	C_1621_H_2594_N_450_O_472_S_14_	91.46
PCP022264.1	3	198	22,669.10	6.75	−0.189	C_1020_H_1585_N_279_O_287_S_10_	88.59
PCP038995.1	2	449	51,146.19	9.93	−0.582	C_2268_H_3685_N_667_O_648_S_15_	80.94
Pm003761	1	350	40,355.22	6.12	−0.456	C_1797_H_2835_N_493_O_531_S_16_	85.2
Pm029730	1	339	38,174.59	5.91	−0.377	C_1694_H_2689_N_467_O_510_S_13_	86.58
Pm021475	3	362	41,251.05	4.92	−0.298	C_1822_H_2864_N_490_O_557_S_22_	88.29
Pm026453	3	362	41,218.18	4.73	−0.228	C_1809_H_2849_N_485_O_556_S_29_	85.88
Pm002088	2	351	40,039.80	5.55	−0.251	C_1790_H_2802_N_480_O_530_S_16_	91.11
Pm004465	2	338	38,359.05	6.43	−0.283	C_1706_H_2689_N_471_O_499_S_18_	86.18
Pm004466	2	520	57,750.25	5.86	−0.058	C_2598_H_4020_N_684_O_753_S_27_	87.9
Prupe.1G573300.1	1	350	40,256.90	6.17	−0.48	C_1792_H_2821_N_497_O_532_S_13_	84.94
Prupe.5G054500.1	1	339	38,170.54	5.91	−0.367	C_1694_H_2689_N_469_O_510_S_12_	87.43
Prupe.8G115900.1	3	362	41,037.62	4.92	−0.306	C_1815_H_2849_N_491_O_557_S_18_	89.39
Prupe.7G138200.1	3	362	40,942.64	4.77	−0.233	C_1802_H_2836_N_486_O_556_S_23_	88.07
Prupe.6G192200.1	2	339	38,550.04	5.46	−0.273	C_1717_H_2702_N_464_O_514_S_15_	92.6
Prupe.1G521600.1	2	338	38,235.89	6.64	−0.273	C_1702_H_2680_N_472_O_496_S_17_	86.48
Prupe.1G521800.1	2	360	40,832.18	6.71	−0.145	C_1834_H_2887_N_495_O_524_S_18_	92.56
Ro05_G31301	1	352	40,450.04	6	−0.525	C_1795_H_2824_N_500_O_537_S_14_	82.5
Ro02_G35920	3	325	36,669.63	4.79	−0.227	C_1631_H_2528_N_432_O_496_S_17_	87.6
Ro01_G29721	3	310	35,087.95	4.7	−0.354	C_1544_H_2436_N_414_O_480_S_19_	87.1
Ro02_G15248	3	286	32,276.65	4.68	−0.36	C_1424_H_2244_N_378_O_446_S_15_	88.95
Ro05_G30878	1	242	27,245.30	8.22	−0.411	C_1208_H_1946_N_334_O_363_S_9_	89.01

### Evolutionary expansion and synteny analysis of *SnRK2* genes in Rosaceae

**Fig. 4:**
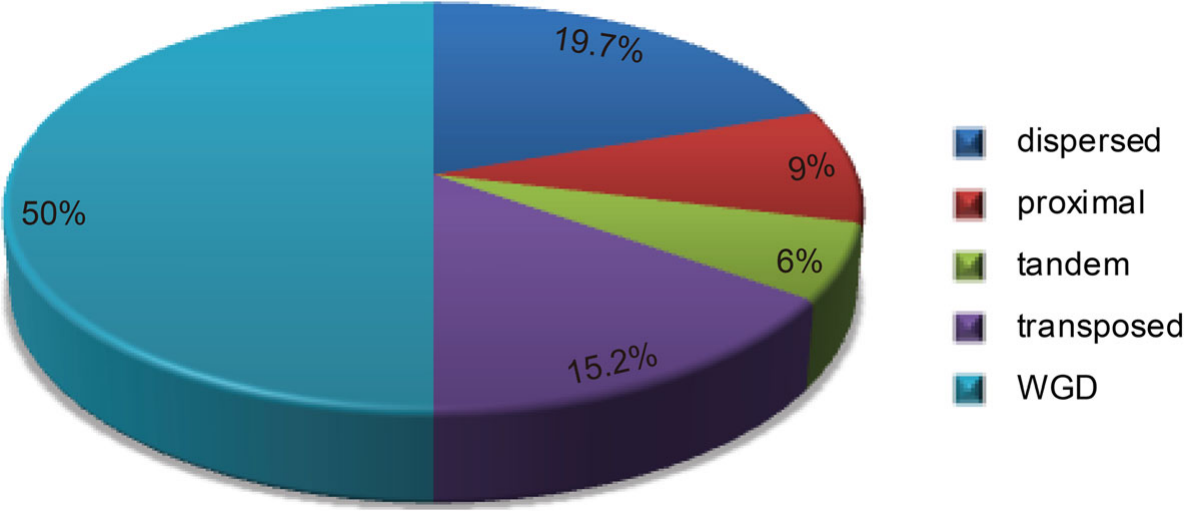
Number of *SnRK2* genes from different origins in eight Rosaceae genomes

Several gene duplication patterns drive the evolution of protein-coding gene families, which include whole-genome duplication (WGD) or segmental duplication, tandem and segmental duplications, and rearrangements at the gene and chromosomal levels [27]. The origins of duplicated genes were explored in the *SnRK2* gene family in eight Rosaceae genomes using the MCScanX package. Each member of the *SnRK2* gene family was allocated to one of five different categories: WGD or segmental, singleton, proximal, tandem, or dispersed. Five types of duplication events contributed to the expansion of the *SnRK2* gene family in Rosaceae: 50% WGD, 19.7% dispersed, 15.2% transposed, 9% proximal, and 6% tandem (Fig. 4). Among them, WGD events occurred in each of the Rosaceae species; in particular, 60% of the *SnRK2* genes in Chinese white pear, 86% in apple, 57.1% in peach, and 50% in strawberry were duplicated and retained from WGD events compared with only 40% in black raspberry, 28.6% in Chinese plum, 28.6% in cherry, and 7.7% in European pear (Table S2). Hence, WGD may have impacted the evolution of the *SnRK2* gene family in Chinese white pear, apple, peach and strawberry. In addition, the proportions of dispersed *SnRK2* gene duplication events in black raspberry (40%), European pear (30%), peach (28.6%), strawberry (28.6%), cherry (28.6%), and apple (7%) were assessed (Table S2). Transposed events were 10% in pear, 37.5% in strawberry, 28.6% in Chinese plum, 28.6% in cherry and 15.4% in European pear. These results indicate that transposed, WGD, and dispersed events impacted the evolution of the *SnRK2* gene family in Chinese plum and cherry. In black raspberry, WGD and dispersed events were the main forces, while dispersed and transposed events played key roles in the evolution of European pear.

**Fig. 5:**
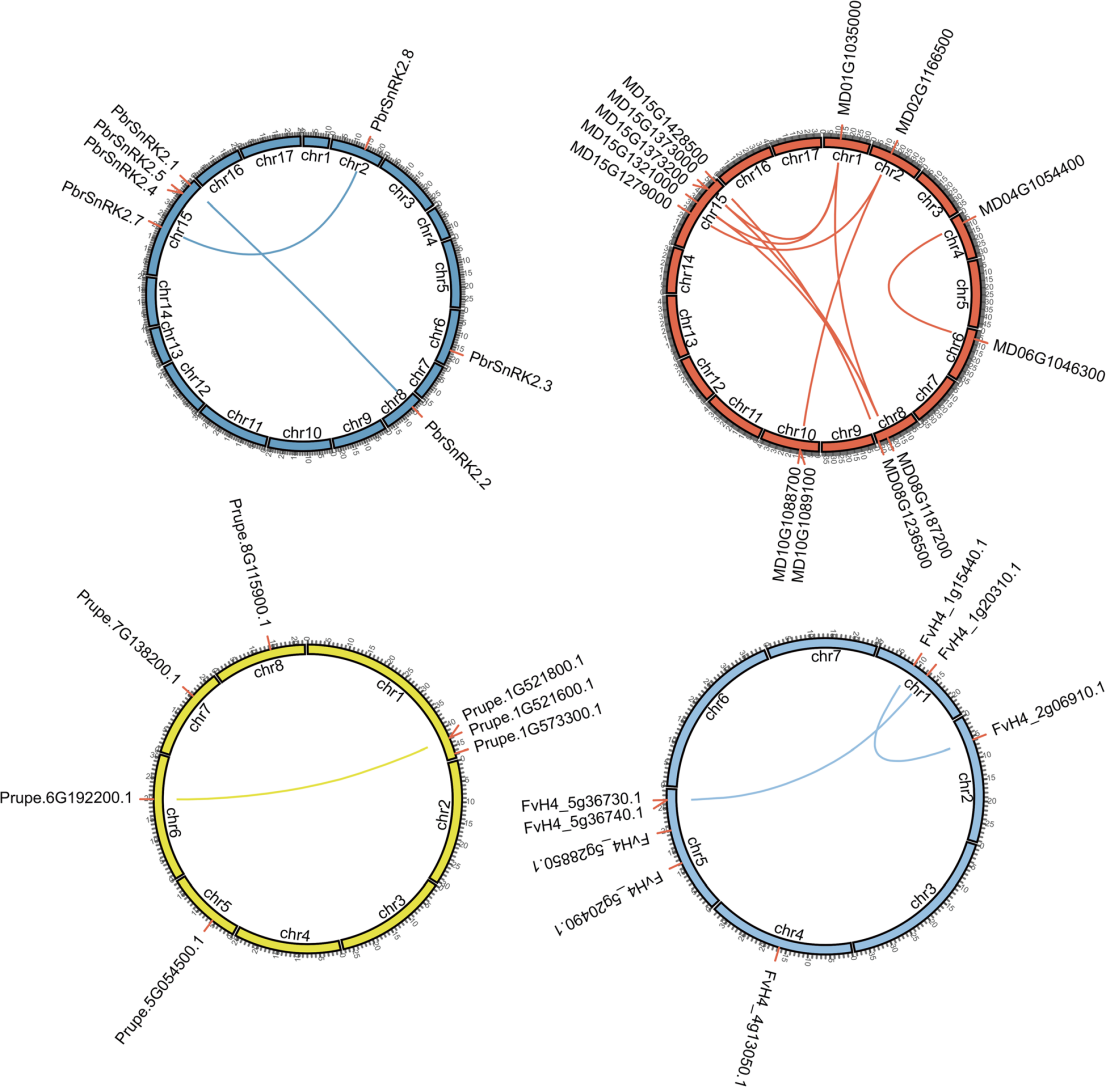
Chromosomal localization and synteny of the *SnRK2* genes in Rosaceae genomes. *SnRK2* genes in Chinese white pear, apple, peach, and strawberry were mapped onto the different chromosomes. Chromosome number is indicated on the inner side in the inner circle, corresponding to different *SnRK2* genes. Gene pairs with a syntenic relationship are joined by a line

To explore the evolutionary process of *SnRK2* genes, an intra-genomic synteny map was constructed for each analyzed Rosaceae specie in the study. The result showed that *PbrSnRK2* genes were distributed on 4 out of the 17 pear chromosomes, with 4 *SnRK2* genes anchored on chromosome 15, and 2 syntenic pairs were identified (Fig. 5). Thirteen *SnRK2* genes were assigned to 7 of the 17 chromosomes in apple, with 5 genes anchored to chromosome 15, and 9 syntenic pairs were identified. Furthermore, 7 *SnRK2* genes were distributed on 5 of the 8 chromosomes in peach, with 3 genes anchored to chromosome 1, and 1 syntenic pair was identified (Fig. 5). Eight *SnRK2* genes were assigned to 4 of the 7 chromosomes in strawberry, with 4 genes anchored to chromosome 5, and 2 syntenic pairs were identified (Fig. 5). In addition, 7 *SnRK2* genes were located on Chr1, Chr2, Chr4, Chr6 and Chr8 in Chinese plum, of these, 3 genes were co-located on Chr2, and 1 syntenic pair was identified (Fig. S2). Five *SnRK2* genes were located on Chr1, Chr2 and Chr5 in black raspberry, of these, 2 genes were co-located on Chr2 and Chr5, respectively, and 1 syntenic pairs was identified (Fig. S2). Seven *SnRK2* genes were located on Chr1, Chr5, Chr6, Chr7 and Chr8 in cherry, of these, 3 genes were co-located on Chr1 and 1 syntenic pairs was identified (Fig. S2). Nine *SnRK2* genes were located on Chr1, Chr2, Chr4, Chr8 and Chr15 in European pear, of these, 4 genes were co-located on Chr15 and 2 genes were co-located on Chr8, and 2 syntenic pairs were identified (Fig. S2).

### Ks value and Ka/Ks ratio reveal dates and driving forces of evolution

**Fig. 6:**
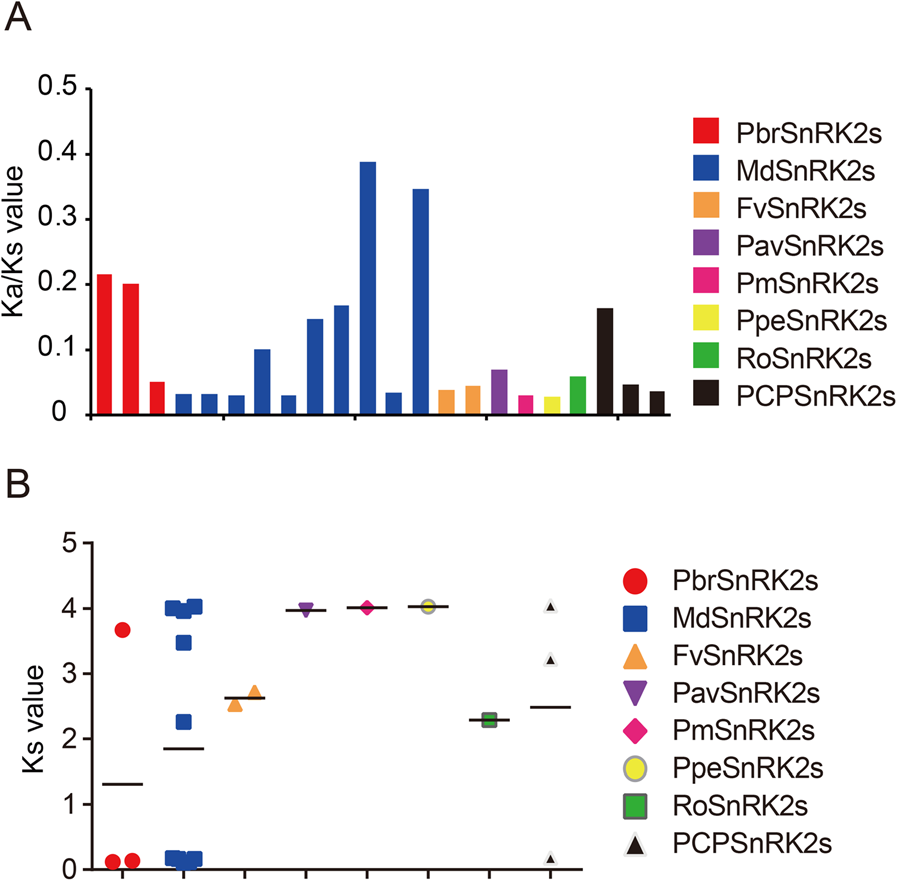
Distribution of mean Ka/Ks and Ka values of *SnRK2* genes in Rosaceae. **a** Ks values indicating the times of *SnRK2* gene evolution in Rosaceae. **b** Ka/Ks ratios indicating the driving forces of *SnRK2* gene evolution in Rosaceae

Purifying selection (negative selection) is the process via which disadvantageous mutations are removed, whereas Darwinian selection (positive selection) accumulates new advantageous mutations and spreads them throughout the population [27]. To identify the selection process that drove the evolution of the *SnRK2* gene family, the Ka value and Ka/Ks ratio of its paralogs were examined in the eight Rosaceae species based on coding sequences. We found that all values were < 1 in the studied Rosaceae species (Fig. 6a), implying that this family underwent a purifying selection pressure during its evolution in Rosaceae and that its evolution was very conservative.

The Ks value is extensively used to evaluate the evolutionary dates of WGD or segmental duplication events. Previous studies have shown that the genome of pear and apple have undergone two genome-wide duplication events (ancient and recent). In Chinese white pear, the recent WGD event that is inferred to have occurred 30–45 MYA (Ks ~ 0.15–0.3) and the ancient WGD event that is inferred to take place ~ 140 MYA (Ks ~ 1.5–1.8) [24]. Furthermore, the recent WGD event that is inferred to have occurred 30–45 MYA (Ks ~ 0.2) and the ancient WGD event that is inferred to take place ~ 140 MYA (Ks ~ 1.6) in apple [28, 29]. To explore the evolutionary dates of the duplication events among the *SnRK2* gene family members, Ks values were analyzed in the Rosaceae species. The results showed that the Ks values for the *SnRK2* gene pairs ranged from 0.107 to 4.0487 in Rosaceae; moreover, the Ks values of WGD gene pairs *PbrSnRK2.1*–*PbrSnRK2.2* (Ks, ~ 0.1028), *PbrSnRK2.7*–*PbrSnRK2.8* (Ks, ~ 0.1363), *PCP022180.1*–*PCP002062.1* (Ks, ~ 0.182), *MD01G1035000*–*MD15G1321000* (Ks, ~ 0.1644), *MD02G1166500*–*MD15G1279000* (Ks, ~ 0.1757), *MD04G1054400*–*MD06G1046300* (Ks, ~ 0.1635), *MD08G1187200*–*MD15G1373000* (Ks, ~ 0.1138), and *MD08G1236500*–*MD15G1428500* (Ks, ~ 0.107) are close to the Ks peak corresponding to the recent WGD that was detected in pear genome, indicating that some *SnRK2* genes were derived and retained from recent WGD events (30–45 MYA) [24]. Furthermore, other duplicated gene pairs (such as *PbrSnRK2.5* and *PbrSnRK2.6*) possessed higher Ks values (2.26–4.0487), indicating that they might have stemmed from a more ancient duplication event (Fig. 6b).

### Expressions of *PbrSnRK2* genes in different tissues

To understand the expression patterns and functional properties of *SnRK2* genes in different tissues, we constructed a heat map at the transcriptional level using MeV to depict the overall expression patterns of *SnRK2* genes in pear based on the transcriptome data from pear root, stem, leaf, fruit, petal, sepal, ovary, bud, pollen, pollen tube and stop-growth pollen tube. The transcriptome data was obtained from previous studies conducted by our group [30–32]. The results showed that *SnRK2* genes were expressed in most organizations of pear, i.e., three genes (*PbrSnRK2.2*/*2.5*/*2.7*) were exhibited high expression in fruits, two (*PbrSnRK2.3*/*2.8*) in leaves, and one (*PbrSnRK2.4*) in roots (Fig. S3). Furthermore, *PbrSnRK2.1* and *PbrSnRK2.6* were highly expressed in petal, *PbrSnRK2.9* was highly expressed in bud, and *PbrSnRK2.10* was highly expressed in ovary.

**Fig. 7:**
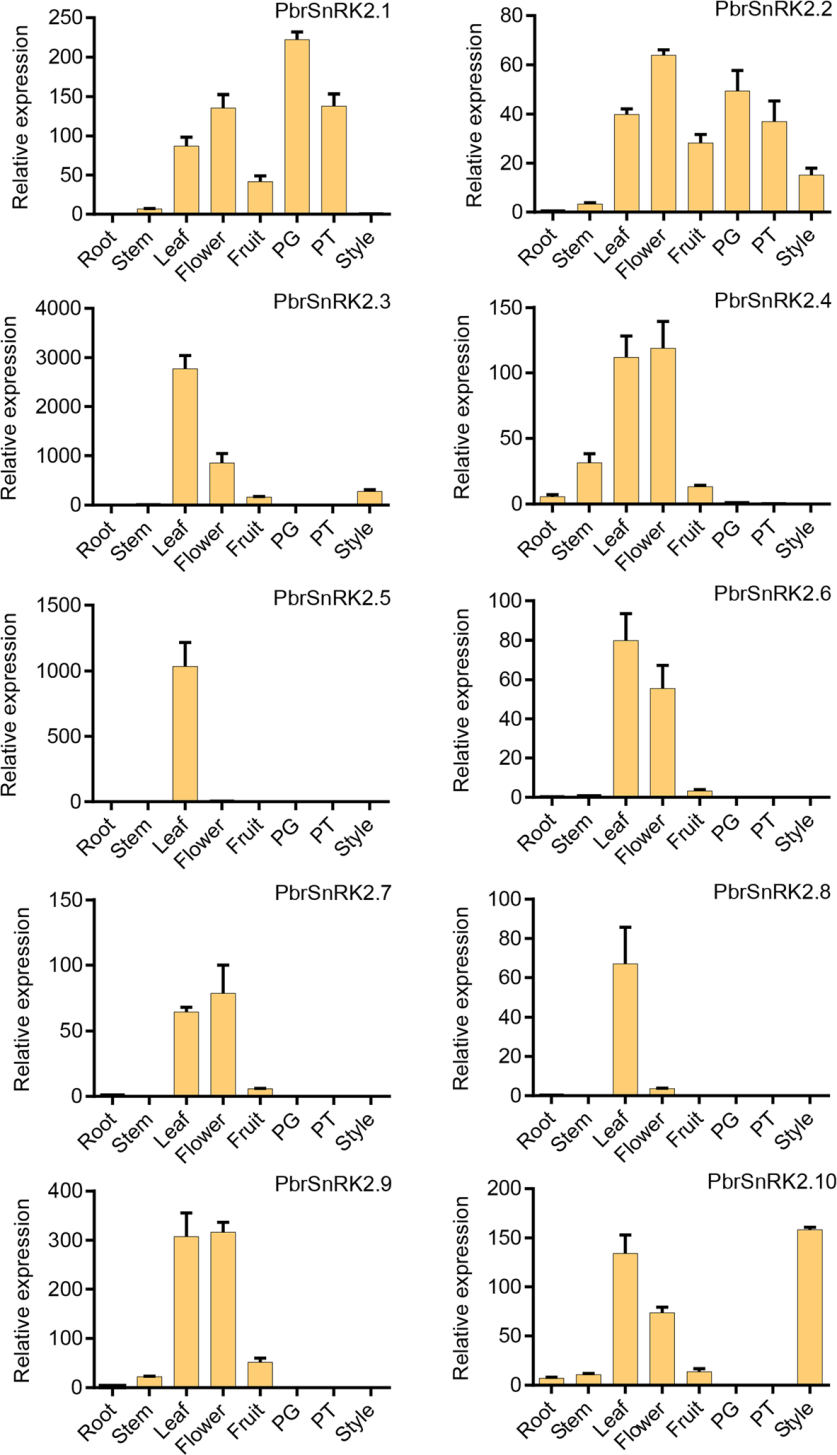
Expression analyses of 10 *PbrSnRK2* genes in different tissues. The relative transcript abundances of *PbrSnRK2* genes were examined by qRT-PCR. Total RNA was extracted from roots, stems, leaves, flowers, fruits, styles, pollen grains, and pollen tubes cultured for 5 h. Regarding each gene, the relative expression levels were obtained by normalization to that of pear *Actin*. The error bars indicate standard deviations. Data are expressed as mean ± standard deviation

In addition, those *PbrSnRK2* genes were also examined by qRT-PCR using gene-specific primers and in diverse pear tissues including root, stem, leaf, flower, fruit, PG, PT and style (Table S3). The results showed that *SnRK2* genes have diverse expression patterns in different pear tissues, although some genes have the same expression pattern between the two methods, and some genes have different expression characteristics in different tissues. For example, *PbrSnRK2.3* and *PbrSnRK2.8* were also highly expressed in leaf, and *PbrSnRK2.6* also exhibited high expression in the flower, which is consistent with transcriptome data (Figs. 7 and S3). *PbrSnRK2.2*, *PbrSnRK2.4* and *PbrSnRK2.7* were highly expressed in flower based on the data of qRT-PCR, nevertheless transcriptome data showed that *PbrSnRK2.2*, *PbrSnRK2.4* and *PbrSnRK2.7* were highly expressed in fruit, root and root, respectively (Figs. 7 and S3). *PbrSnRK2.5* and *PbrSnRK2.9* were highly expressed in leaf by qRT-PCR; *PbrSnRK2.1* was highly expressed in PG, and *PbrSnRK2.10* was highly expressed in style by qRT-PCR (Fig. 7). These results indicated that *SnRK2* genes may play important roles in these pear tissues and respond to abiotic in different organizations. In addition, these genes may have different expression characteristics in different developmental stages of plant tissues.

### Expression profiles of *PbrSnRK2* genes under ABA treatment

**Fig. 8:**
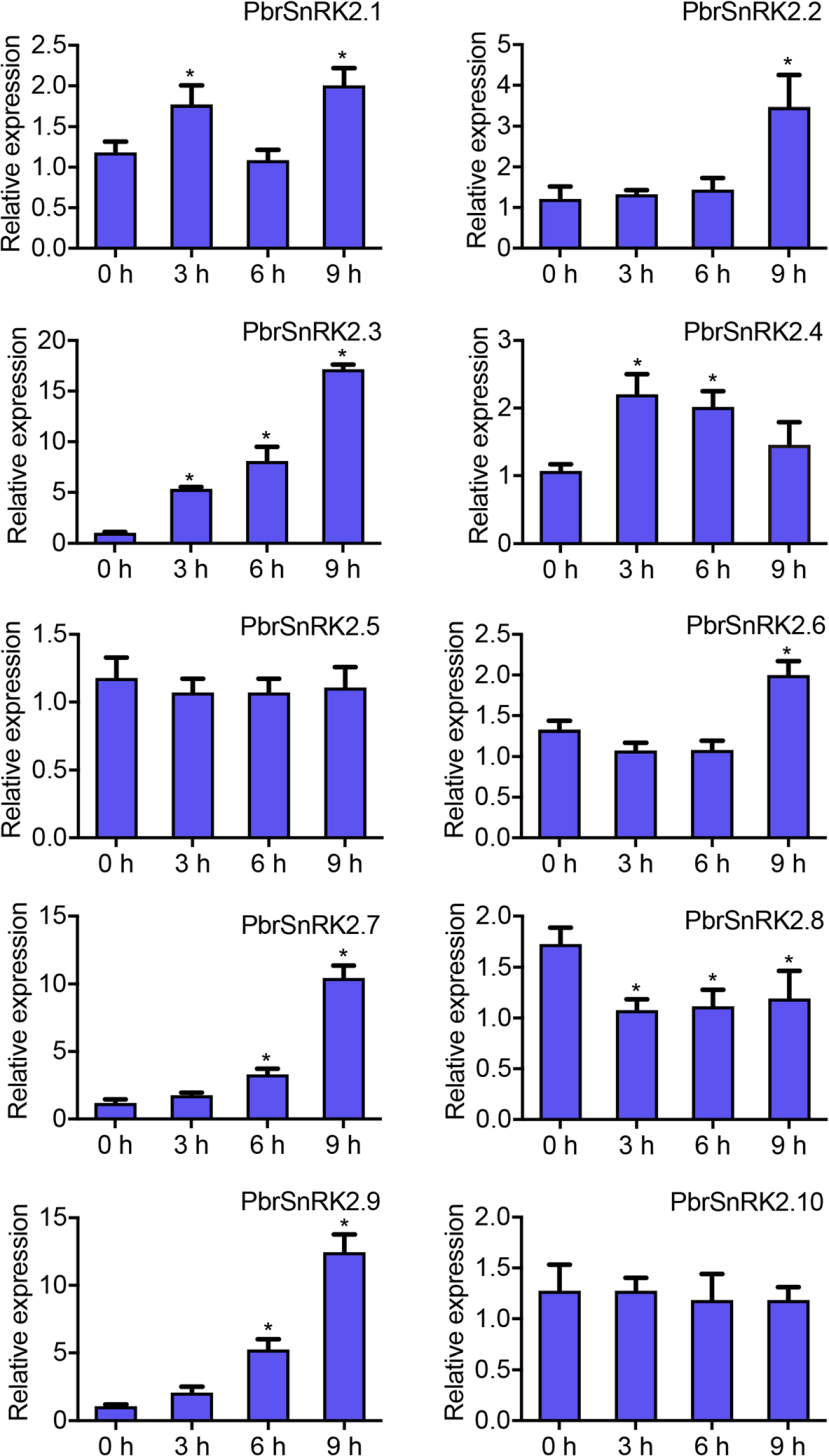
Expression patterns of *PbrSnRK2* genes under abiotic treatments. The relative transcript levels of *PbrSnRK2.1*–*PbrSnRK2.10* were examined by qRT-PCR, and the samples were harvested at 0, 3, 6, and 9 h after foliar spraying in leaves from 3-month-old seedlings under 50 μM ABA stress treatments. Regarding each gene, the relative expression levels were obtained by normalization to that of pear *Actin*. The error bars indicate standard deviations. Asterisks indicate a significant difference (*P < 0.05) compared with 0 h at the different time points after ABA treatment. Data are expressed as mean ± standard deviation

Many studies have shown that SnRK2s play key roles in response to multiple abiotic stresses such as salinity, dehydration, and hyperosmotic stress [14]. Moreover, the members of the *SnRK2* gene family are involved in the regulation of phytohormone pathway responses, particularly ABA signal transduction [4]. To explore the dynamic transcriptional changes in *PbrSnRK2* genes in response to ABA treatment, the expression levels of these genes in leaves of pear were evaluated by qRT-PCR under ABA (50 μM) treatment at four time points: 0, 3, 6, and 9 h. The results showed that in response to exogenous ABA application, the *SnRK2* genes exhibited different expression patterns. For example, eight genes (*PbrSnRK2.1/2.2/2.3/2.4/2.6/2.7/2.8/2.9*) were activated by ABA, whereas the expressions of two genes (*PbrSnRK2.5/10*) remained unchanged at each time point (Fig. 8). Among them, the expression levels of *PbrSnRK2.1*, *PbrSnRK2.3*, and *PbrSnRK2.4* were significantly upregulated at 3 h after ABA treatment, and the expression pattern of *PbrSnRK2.1* shows a wavy trend with ABA treatment (Fig. 8), whereas *PbrSnRK2.4*’s expression pattern shows a parabolic trend under ABA treatment. *PbrSnRK2.7* and *PbrSnRK2.9* showed similar expression patterns. Specifically, they were both significantly upregulated at 6 h after ABA treatment, and reached their peak value at 9 h after ABA treatment (Fig. 8). The expression levels of *PbrSnRK2.2/2.6* were significantly upregulated at 9 h after ABA treatment. However, *PbrSnRK2.8* was downregulated over time after ABA treatment (Fig. 8).

## Discussion

SnRK2 is a specific family of Ser/Thr protein kinases in plants that play important roles in the transduction of various signaling pathways, particularly in response to abiotic stress [4, 5]. With the completion of whole-genome sequencing in an increasing number of organisms, it has become possible to comprehensively analyze and study the functions of gene families from the genomic perspective. The members of the *SnRK2* gene family have been identified and analyzed in many plant species [33, 34]; however, a comprehensive systematic investigation of this family remains limited in Rosaceae. Here, 71 *SnRK2* genes were identified in Rosaceae and the same number of *SnRK2* genes was identified in pear, compared with 10 in *Arabidopsi*s. The *SnRK2* gene families in Chinese white pear, apple, and European pear have more members compared with those in cherry, strawberry, and black raspberry. Recent genome-wide evidence revealed that pear and apple have undergone both a recent WGD event and an ancient WGD event; however, strawberry, peach, Chinese plum, black raspberry, and cherry did not undergo this event [27]. Our results, which showed that pear and apple experienced two WGD event (recent and ancient), also support the conclusion. Therefore, there is a possibility that WGD event lead to the number differentiation of *SnRK2* genes detected in the investigated Rosaceae species. Moreover, these *SnRK2* genes were classified into three subfamilies, subgroups I, II, and III, which is consistent with the classification of other *SnRK2* genes [15, 16]. In addition, the conserved motif and exon-intron analysis also support the classification of *SnRK2* genes in pear and *Arabidopsis*, regardless of the loss of exon-intron or motif in some *PbrSnRK2* genes. We believe that the loss may be due to the structural polymorphism of the *PbrSnRK2* genes in the long-term evolution process, simultaneously resulting in some functional differentiation.

To investigate the evolutionary process and history of *SnRK2* genes in Rosaceae and to explore further their functions in response to various abiotic stresses, gene duplication events, gene syntenic relationships, and Ka/Ks values of *SnRK2* genes were investigated in Rosaceae. Dispersed, tandem, proximal, transposed, and genome-wide duplications differentially contribute to the expansion of specific gene families in plant genomes [35]. The results of the synteny analysis performed here indicated that the *SnRK2* genes in Chinese white pear, apple, peach and strawberry were derived primarily from WGD events. Dispersed duplications were the major drivers of *SnRK2* gene family expansion in European pear. Furthermore, the expansion of the *SnRK2* gene family underwent five types of duplication events, with WGD events accounting for 50% of the *SnRK2* genes in Rosaceae. Among these events, four duplicated gene pairs, i.e., *PbrSnRK2.1*–*PbrSnRK2.2*, *PbrSnRK2.7*–*PbrSnRK2.8*, *MD08G1236500*–*MD15G1428500*, and *MD08G1187200*–*MD15G1373000*, had lower Ks values (0.12, 0.13, 0.11, and 0.11, respectively), suggesting that they resulted from a recent duplication event. However, the Ks values of other duplicated gene pairs, i.e., *MD08G1187200*–*MD15G1321000* and *PbrSnRK2.5*–*PbrSnRK2.6*, and *pm02088*–*pm004465*, were > 3, which indicated that they resulted from a more ancient duplication event. This is similar to previous observation, i.e., > 90% of the increase in functional genes in the *Arabidopsis* lineage is due to expansion resulting from genome duplication events [36]. These results indicate that WGD events may play a key role in the expansion of the *SnRK2* gene family and that *SnRK2* genes function in response to various biological and abiotic stresses. This result is similar to previous observations in studies that analyzed the expansion of the *Hsf* and *F-box* gene family in pear [27, 37].

Purifying selection (negative selection) is the process via which deleterious mutations are eliminated. In contrast, Darwinian selection (positive selection) accumulates new advantageous mutations and spreads them across the population [38]. The Ka/Ks ratio was further calculated for exploring the selection process that drove the evolution of the *SnRK2* gene family in Rosaceae. In this study, the Ka/Ks ratios of all paralogous genes were < 0.5. Previous studies have shown that the Ka/Ks ratio represents selection magnitude and direction measure: 1 indicates neutral evolution, < 1 indicates negative selection, and > 1 indicates positive selection [39]. These results indicate that negative selection may play a key role in the evolution of these *SnRK2* genes.

Gene expression patterns can provide important clues for describing gene function. Therefore, information pertaining to the specific expressions of *PbrSnRK2* genes was analyzed in various tissues using public RNA-Seq datasets and qRT-PCR. The results revealed that *PbrSnRK2* genes exhibited diverse spatiotemporal expression patterns in different tissues of pear. For example, the qRT-PCR results showed that *PbrSnRK2.3*, *PbrSnRK2.5*, and *PbrSnRK2.8* were preferentially expressed in leaves, implying that the proteins encoded by them possibly participate in leaf development and stomatal regulation in response to abiotic stress. The expression patterns of these genes were similar to those of *AtSnRK2.3* in *Arabidopsis,* which was also highly expressed in leaves [40]. In addition, researchers have shown that *AtSnRK2.3* is mainly involved in the water stress response of ABA signaling in leaves [41]. Conversely, *PbrSnRK2.1*, *PbrSnRK2.2*, *PbrSnRK2.4*, *PbrSnRK2.7*, *PbrSnRK2.9*, and *PbrSnRK2.10* exhibited strong expression in germ cells such as flowers, pollen, and styles, thereby implying that their encoded proteins potentially participate in the reproductive growth of plants and adaptation to various adverse environments. The expression patterns of these genes were similar to those of *AtSnRK2.7* and *AtSnRK2.9* in *Arabidopsis*, which were also highly expressed in inflorescence [40]. Nevertheless, further studies are necessary to verify whether PbrSnRK2s play a key role in leaf development and the reproductive growth of plants in response to various abiotic stresses. Furthermore, previous studies have shown that SnRK2s exhibit widespread expressions in various tissues of *Arabidopsis* as well as are involved in seed germination and growth [42], regulation of primary metabolism [7] and stomatal aperture [43]. For example, *AtSnRK2.2* is expressed in both seeding and cell suspension [44], and *AtSnRK2.8* is mainly expressed in roots [45], whereas *AtSnRK2.6* is highly expressed in stems and roots [7]. Furthermore, the expression patterns of *SnRK2* genes have been reported in other species such as *MpSnRK2.4*, which is mainly expressed in fruit and may participate in fruit development in apple [23]; *HbSnRK2.2*, which is high expressed in leaf and flower tissues [15]; and *StSnRK2.4*, which is mainly expressed in shoot tissues and may be involved in responses to stress signaling [46].

Many studies have shown that *SnRK2* genes play a key role in response to various environmental stresses such as salinity, low temperature, and drought [34]. Moreover, *SnRK2* genes play important roles in the regulation of phytohormones, particularly of ABA signal transduction [33]. In this study, the amino acid sequences of SnRK2 proteins were aligned in pear against those in *Arabidopsis*. The result showed that Domain I exists in each SnRK2 protein, which is consistent with previous study that Domain I is necessary in all SnRK2s and is needed for activation by osmotic stress [34]. Nevertheless, Domain II in subgroup III only exists in strong ABA-responsive kinases [34], and the results of verification assay accorded with this conclusion, i.e. *PbrSnRK2.7* and *PbrSnRK2.9* in subgroup III were strongly stimulated by ABA. Contrary to earlier reports, we found that *PbrSnRK2.3* in the subgroup III was also strongly stimulated by ABA, and Domain II existed in PbrSnRK2.3, PbrSnRK2.7 and PbrSnRK2.9. Possible reason for these phenomenon is that *SnRK2* gene family had expanded and displayed the structural diversity in a subgroup, which led to functional differentiation [47]. Furthermore, we also explored the dynamic expression levels of *PbrSnRK2* genes in pear by treating the leaves with ABA. The results expanded our understanding of the functions of PbrSnRK2s in response to phytohormones and showed that all *PbrSnRK2s* but *PbrSnRK2.5* and *PbrSnRK2.10* were induced at different time points after ABA treatment. For example, *PbrSnRK2.1*, *PbrSnRK2.3*, and *PbrSnRK2.4* were upregulated at 3 h after ABA treatment, similar to previous findings of *StSnRK2.2*, *StSnRK2.4*, *GhSnRK2.7* and *MpSnRK2.7* responding quickly to ABA treatment at 3 h in leaves [23, 46, 48]. *PbrSnRK2.7*, and *PbrSnRK2.9* were upregulated at 6 h after ABA treatment, which is similar with the expression promotion of *StSnRK2.3*, *HbSnRK2.5/2.7/2.8/2.9/2.10*, *MpSnRK2.1*, and *MpSnRK2.2* in leaves after ABA treatment [15, 23, 46]. Among them, the expression levels of *PbrSnRK2.3*, *PbrSnRK2.7*, and *PbrSnRK2.9* were significantly induced at 9 h after ABA treatment. Moreover, *PbrSnRK2.2*, and *PbrSnRK2.6* were also upregulated at 9 h after ABA treatment, similar with previous studies that *HbSnRK2.9* responds to ABA treatment at 9 h in leaves [15]. In contrast, the expression levels of *PbrSnRK2.8* was downregulated at 3 h after ABA treatment, and this gene similar to *StSnRK2.1/2.5/2.7* expression pattern, which were downregulated significantly in leaves after ABA treatment [46]. These results indicate that the response of most *SnRK2* genes to the various abiotic stresses is achieved via the ABA signaling pathway, which may be particularly involved in the regulation of salt stress and drought stress tolerance in pear. However, additional investigation is needed regarding the functional characterization of PbrSnRK2s. Furthermore, *SnRK2* genes reportedly respond to various abiotic stresses in other species. Many of the *SnRK2* genes (such as *SAPK8*, *SAPK9*, and *SAPK10*) were differentially regulated by ABA in different organs of rice [18]. The expression levels of *TaSnRK2.7* and *TaSnRK2.8* genes from wheat were induced in response to ABA treatment [4]. *MpSnRK2.8*, *MpSnRK2.9*, and *MpSnRK2.10* exhibited strong responses to ABA application in apple [23]. In *Arabidopsis*, *AtSnRK2.2*, *AtSnRK2.3*, and *AtSnRK2.6* are typically activated by ABA treatment, which can phosphorylate the ABA-responsive elements [20].

## Conclusion

In this study, 71 *SnRK2* genes were identified and characterized in eight Rosaceae species; among them, 10 SnRK2 proteins were identified in Chinese white pear. The structural characteristics of the encoded proteins and phylogenetic analysis showed that the *SnRK2* gene family was clustered into three well-supported clades (Groups I, II, and III) and that most of the members of the PbrSnRK2 family had two conserved kinase domains in the N-terminal and C-terminal regions. In addition, synteny analyses revealed that SnRK2 genes underwent a purifying selection pressure during the evolution of Rosaceae and that WGD and dispersed duplication played key roles in the expansion of the SnRK2 gene family in Rosaceae. The relative expressions of the 10 SnRK2 genes varied among tissues and abiotic stresses, with prolonged ABA treatments showing that PbrSnRK2s are expressed in different tissues and respond to various abiotic stresses via the ABA signaling pathway. Our results represent a foundation for further studies that will dissect the structures and functions of the SnRK2 gene family in Rosaceae.

## Methods

### Identification of SnRK2 gene family members in Rosaceae

To identify the *SnRK2* genes in Rosaceae species, multiple database searches were performed. The amino acid sequences of AtSnRK2s were downloaded from The *Arabidopsis* Information Resource (http://www.arabidopsis.org/, TAIR, 10) and the rice SnRK2 amino acid sequences were downloaded from Phytozome (http://phytozome.jgi.doe.gov/pz/portal.html#, *v*9.1). These amino acid sequences were used as queries in BLAST algorithm-based searches against SnRK2 sequences. The genome sequence of pear (*Pyrus bretschneideri*, NJAU, *v*1.1) was downloaded from the Pear Genome Project (http://peargenome.njau.edu.cn/) [24]. Genome sequences of peach (*Prunus persica*, JGI, *v*2.1) and apple (*Malus domestica*, JGI, *v*1.1) were obtained from the Joint Genome Institute (JGI, http://www.jgi.doe.gov/). Genome sequences of strawberry (*Fragaria vesca*, GDR, *v*4.0), black raspberry (*Rubus occidentalis*, GDR, *v*3.0), European pear (*Pyrus communis*, GDR, *v*1.1) and sweet cherry (*Prunus avium*, GDR, *v*1.0) were obtained from the Genome Database for Rosaceae (GDR, http://www.Rosaceae.org/). In addition, the Chinese plum (*Prunus mume*, BUF, *v*1.0) genome sequence was downloaded from National Center for Biotechnology Information (NCBI, https://www.ncbi.nlm.nih.gov/assembly/GCF_000346735.1, *v*1.0). Furthermore, the seed alignment data for the SnRK2 domain (PF00069.25) obtained from the Pfam database were used to build an HMM file using the HMMER3 software package [49]. HMM searches were then performed against the local protein databases of Rosaceae using HMMER3. Finally, all obtained SnRK2 amino acid sequences were checked for the presence of SnRK2 family domains using Pfam (http://pfam.xfam.org) and SMART (http://smart.embl-heidelberg.de/); subsequently, sequences lacking the SnRK2 domain or with E-values of >e^− 10^ or redundant sequences were removed.

### Sequence alignment and phylogenetic and structural analyses

Multiple sequence alignment of SnRK2 proteins in pear and *Arabidopsis* was performed using DNAMAN software. The program SCANPROSITE (http://www.prosite.expasy.org/scanprosite/) was used to detect the protein kinase conserved domains. Phylogenetic trees were constructed based on the SnRK2 amino acid sequences from *Arabidopsis*, rice, and Rosaceae species using the maximum likelihood method in MEGA6.0 (http://www.megasoftware.net/) [50], with amino acid sequences aligned using MUSCLE. The bootstrap test was performed with 1000 replicates, and p-distance and pairwise deletion option parameters were selected. Furthermore, based on the cDNA sequences and their corresponding genomic DNA sequences, the structures of the *SnRK2* genes were analyzed using the Gene Structure Display Server v2.0 (http://gsds.cbi.pku.edu.cn/). The conserved motifs of SnRK2 protein were identified using the Multiple Expectation-Maximization for Motif Elicitation (MEME) tool version 5.0.5 (http://meme-suite.org/tools/meme) using full-length amino acid sequences [51].

### Chromosomal location, synteny analysis, and calculation of the Ka and Ks values

The information of chromosomal localization of *SnRK2* genes was obtained from genome annotation documents [24]. A method similar to that developed for PGGD (http://chibba.agtec.uga.edu/duplication/) was then performed for synteny analysis [52]. Initially, BLASTP was used to search for potential homologous gene pairs (E < 1 e^− 6^) across multiple genomes. Subsequently, MCScanX was used to identify syntenic chains using homologous pairs as input [53]. In addition, MCScanX was used to identify tandem, whole-genome/segmental, proximal, and dispersed duplications in the *SnRK2* gene family. The data were then plotted using Circos software [54]. The Ka and Ks substitution rates of syntenic gene pairs were identified by the MCScanX downstream analysis tools. The mean Ks values of orthologous *SnRK2* gene pairs among Rosaceae species were calculated using all homologous gene pairs located in the same syntenic block. KaKs Calculator 2.0 was used to confirm the Ka and Ks values [27].

### Plant treatments and qRT-PCR assays

To examine the expression patterns of *PbrSnRK2* genes in response to abiotic stresses, the leaves of 3-month-old pear seedlings were sprayed with 50 μM ABA solutions. Samples were harvested from leaves at 0, 3, 6, and 9 h after treatment. All samples were frozen immediately in liquid nitrogen and maintained at − 80 °C for total RNA extraction. Total RNA was isolated independently from roots, stems, leaves of 3-month-old pear seedlings, flowers (8-year-old pear tree), ripening fruits (8-year-old pear tree), styles (8-year-old pear tree), pollen grains (8-year-old pear tree), and pollen tubes (8-year-old pear tree) cultured for 5 h as well as samples for ABA treatment using the RNA Extraction Kit (Invitrogen™ TRIzol® Reagent, Thermo Scientific, Nanjing, China), according to the manufacturer’s instructions. Subsequently, the first-strand cDNA was synthesized using M-MLV reverse transcriptase (TaKaRa, Biotech, Shanghai, China). Specific primers for *PbrSnRK2s* and the housekeeping *Actin* gene *Pbr035825.1* in pear were designed using Primer Premier 5.0 (Table S3), and we used the program Primer Search-Paired against the pear genome to verify the specificity of these primers. Three biological and three technical replicates were used for qRT-PCR assays, and each PCR reaction included 10 μL of LightCycler 480 SYBRGREEN I Master Mix (Roche, Basel, Switzerland), 100 ng of cDNA, and 200 nM of each primer in a final volume of 20 μL. All reactions were conducted using the CFX96 Real-Time System (Roche), Relative expression levels were calculated using the 2^–ΔΔCt^ method and normalized to the *PbrSnRK2* genes. The results were analyzed using Office 2010, and statistical analyses were performed with SPSS17.0 using Duncan’s multiple-range test at a significance level (*P*) of < 0.05.

## Supplementary Information

**Additional file 1: Figure S1.** Phylogenetic relationship of conserved motif of *SnRK2* genes from pear and *Arabidopsis thaliana*. A.A phylogenetic tree constructed with CluxtalX2.0 using the full-length amino acid sequences of *SnRK2* genes from pear and *Arabidopsis*. Bootstrap analysis was performed using 1000 replicates. The species in which SnRK2 proteins were functionally characterized are displayed as icons, and the different colors in the branches represent the different subfamilies. B. MEME tools were used to identify conserved motifs of SnRK2 proteins, different colors represent different motifs. The scale bar indicates 50 aa.

**Additional file 2: Figure S2.** Chromosomal localization and synteny of *SnRK2* genes in Rosaceae genomes. *SnRK2* genes in Chinese plum, black raspberry, cherry, and European pear were mapped onto different chromosomes. Chromosome number is indicated on the inner side in the inner circle corresponding to different *SnRK2* genes. Gene pairs with a syntenic relationship are joined by a line.

**Additional file 3: Figure S3.** Analysis of the expression levels of *PbrSnRK2* in different pear tissues. A heat map depicting the overall trend of the differential expression profiles of *PbrSnRK2* genes in different pear tissues was constructed using MeV. The rows in the heat map represent genes and columns represent tissues. The colors of heat map cells indicate the scaled expression levels of genes across different tissues. The color gradient from blue to yellow corresponds to low-to-high transcript levels.

**Additional file 4: Table S1.** Sequence identity of protein sequences between PbrSnRK2s and AtSnRK2s based on pairwise comparison.

**Additional file 5: Table S2.** Number of *SnRK2* genes from different origins in eight Rosaceae genomes.

**Additional file 6: Table S3.** Primers used in this study.

## Data Availability

All data and materials used in this study are publicly available.

## Abbreviations

SnRK2: Sucrose non-fermenting 1-related protein kinase 2
WGD: Whole-genome duplication
ABA: Abscisic acid
PGDD: Plant genome duplication database
qRT-PCR: Quantitative real-time PCR
PG: Pollen grain
PT: Pollen tube
HMM: Hidden Markov Model

## References

[1] Viswanathan C, Karen S, Zhu J (2004). Molecular genetic perspectives on cross-talk and specificity in abiotic stress signalling in plants. J Exp Bot.

[2] Umezawa T, Sugiyama N, Takahashi F, Anderson JC, Ishihama Y, Peck SC, Shinozaki K. Genetics and Phosphoproteomics reveal a protein phosphorylation network in the Abscisic acid signaling pathway in *Arabidopsis thaliana*. Sci Signal. 2013. 10.1126/scisignal.2003509 23572148

[3] Hardie DG (1999). Plant protein serine/threonine kinases: classification and functions. Annu Rev Plant Phys.

[4] Kulik A, Wawer I, Krzywińska E, Bucholc M, Dobrowolska GY (2011). SnRK2 protein kinases-key regulators of plant response to abiotic stresses. OMICS.

[5] Fujita Y, Yoshida T, Yamaguchi-Shinozaki K (2012). Pivotal role of the AREB/ABF-SnRK2 pathway in ABRE-mediated transcription in response to osmotic stress in plants. Physiol Plantarum.

[6] Huai JL, Wang M, He J (2008). Cloning and characterization of the SnRK2 gene family from Zea mays. Plant Cell Rep.

[7] Zheng Z, Xu X, Crosley RA, Greenwalt SA, Sun Y, Blakeslee B, Wang L, Ni W, Sopko MS, Yao C (2010). The protein kinase SnRK2.6 mediates the regulation of sucrose metabolism and plant growth in Arabidopsis. Plant Physiol.

[8] Kim MJ, Park MJ, Seo PJ, Song JS, Kim HJ, Park CM (2012). Controlled nuclear import of the transcription factor NTL6 reveals a cytoplasmic role of SnRK2.8 in the drought-stress response. Biochem J.

[9] McLoughlin F, Galvan-Ampudia CS, Julkowska MM, Caarls L (2012). Does Dvd: the Snf1-related protein kinases SnRK2.4 and SnRK2.10 are involved in maintenance of root system architecture during salt stress. Plant J.

[10] Zhang H, Mao X, Zhang J, Chang X, Jing R (2013). Single-nucleotide polymorphisms and association analysis of drought-resistance gene TaSnRK2.8 in common wheat. Plant Physiol Biochem.

[11] Ying S, Zhang D, Li H, Liu Y, Shi Y, Song Y, Wang TY, Li Y (2011). Cloning and characterization of a maize SnRK2 protein kinase gene confers enhanced salt tolerance in transgenic Arabidopsis. Plant Cell Rep.

[12] Sun SJ, Qi GN, Gao QF, Wang HQ, Yao FY, Hussain J, Wang YF (2016). Protein kinase OsSAPK8 functions as an essential activator of S-type anion channel OsSLAC1, which is nitrate-selective in rice. Planta.

[13] Su Y, Wang Y, Zhen J, Zhang X, Chen Z, Li L, Huang Y, Hua J (2017). SnRK2 homologs in Gossypium and GhSnRK2.6 improved salt tolerance in transgenic upland cotton and Arabidopsis. Plant Mol Biol Rep.

[14] Shao Y, Zhang X, Van Nocker S, Gong X, Ma F (2019). Overexpression of a protein kinase gene MpSnRK2.10 from Malus prunifolia confers tolerance to drought stress in transgenic *Arabidopsis thaliana* and apple. Gene.

[15] Guo D, Li HL, Zhu JH, Wang Y, Peng SQ (2017). Genome-wide identification, characterization, and expression analysis of SnRK2 family in *Hevea brasiliensis*. Tree Genet Genomes.

[16] Wei Z, Cheng YH, Chi Z, Shen XJ, You QB, Wei G, Xiang L, Song XJ, Zhou XA, Jiao YQ (2017). Genome-wide identification and characterization of the GmSnRK2 family in soybean. Int J Mol Sci.

[17] Yoshida R, Umezawa T, Mizoguchi T, Takahashi S, Takahashi F, Shinozaki K (2006). The regulatory domain of SRK2E/OST1/SnRK2.6 interacts with ABI1 and integrates Abscisic acid (ABA) and osmotic stress signals controlling Stomatal closure in Arabidopsis. J Biol Chem.

[18] Kobayashi Y, Yamamoto S, Minami H, Kagaya Y, Hattori T (2004). Differential activation of the rice sucrose nonfermenting1-related protein kinase2 family by hyperosmotic stress and abscisic acid. Plant Cell.

[19] Burza AM, Pekala I, Sikora J, Siedlecki P, Malagocki P, Bucholc M, Koper L, Zielenkiewicz P, Dadlez M, Dobrowolska G (2006). Nicotiana tabacum osmotic stress-activated kinase is regulated by phosphorylation on Ser-154 and Ser-158 in the kinase activation loop. J Biol Chem.

[20] Tian X, Ren R, Zhang YY, Pang Y, Yan C, Gong X, Yuan H, Li W, Di M, Qi H (2011). Molecular mechanism for the inhibition of a critical component in the Arabidopsis thaliana abscisic acid signal transduction pathways, SnRK2.6, by the protein phosphatase ABI1. J Biol Chem.

[21] Zhang H, Mao X, Jing R, Chang X, Xie H (2011). Characterization of a common wheat (Triticum aestivum L.) TaSnRK2.7 gene involved in abiotic stress responses. J Exp Bot.

[22] Monks DE, Aghoram K, Courtney PD, De Wald DB, Dewey RE (2001). Hyperosmotic stress induces the rapid phosphorylation of a soybean phosphatidylinositol transfer protein homolog through activation of the protein kinases SPK1 and SPK2. Plant Cell.

[23] Shao Y, Qin Y, Zou Y, Ma F (2014). Genome-wide identification and expression profiling of the SnRK2 gene family in Malus prunifolia. Gene.

[24] Wu J, Wang ZW, Shi ZB, Zhang S, Ming R, Zhu SL, Khan MA, Tao ST, Korban SS, Wang H (2013). The genome of the pear (*Pyrus bretschneideri* Rehd.). Genome Res.

[25] Chen G, Chen Q, Qi K, Xie Z, Yin H, Wang P, Wang R, Huang Z, Zhang S, Wang L (2019). Identification of shaker K^+^ channel family members in Rosaceae and a functional exploration of PbrKAT1. Planta.

[26] Chen G, Li X, Qiao X, Li J, Wang L, Kou X, Wu X, Wang G, Yin H, Wang P (2019). Genome-wide survey and expression analysis of the SLAC/SLAH gene family in pear (*Pyrus bretschneideri*) and other members of the Rosaceae. Genomics.

[27] Qiao X, Li M, Li LT, Yin H, Wu JY, Zhang SL (2015). Genome-wide identification and comparative analysis of the heat shock transcription factor family in Chinese white pear (*Pyrus bretschneideri*) and five other Rosaceae species. BMC Plant Biol.

[28] Velasco R, Zharkikh A, Affourtit J, Dhingra A, Cestaro A, Kalyanaraman A, Fontana P, Bhatnagar SK, Troggio M, Pruss D (2010). The genome of the domesticated apple (*Malus*× *domestica* Borkh.). Nat Genet.

[29] Fawcett JA, Maere S (2009). Van dP, Y.: plants with double genomes might have had a better chance to survive the cretaceous-tertiary extinction event. Proc Natl Acad Sci.

[30] Zhou H, Yin H, Chen J, Liu X, Zhang S (2016). Gene-expression profile of developing pollen tube of Pyrus bretschneideri. Gene Expr Patterns.

[31] Li Q, Qiao X, Yin H, Zhou Y, Dong H, Qi K, Li L, Zhang S. Unbiased subgenome evolution following a recent whole-genome duplication in pear (*Pyrus bretschneideri* Rehd.). Horticulture Res. 2019;6(34):1–12. 10.1038/s41438-018-0110-6 PMC6395616 30854211

[32] Chen G, Li X, Chen Q, Wang L, Qi K, Yin H, Qiao X, Wang P, Zhang S, Wu J (2018). Dynamic transcriptome analysis of root nitrate starvation and re-supply provides insights into nitrogen metabolism in pear (*Pyrus bretschneideri*). Plant Sci.

[33] Chen S, Jia H, Wang X, Shi C, Li J. Hydrogen sulfide positively regulates abscisic acid signaling through persulfidation of SnRK2.6 in guard cells. Mol Plant. 2020;13(5):732–44. 10.1016/j.molp.2020.01.004 31958520

[34] Lin Z, Li Y, Zhang Z, Liu X, Wang P. A RAF-SnRK2 kinase cascade mediates early osmotic stress signaling in higher plants. Nat Commun. 2020;11(1):1–10. 10.1038/s41467-020-14477-9 PMC6992735 32001690

[35] Maher C, Stein L, Ware D (2006). Evolution of Arabidopsis microRNA families through duplication events. Genome Res.

[36] Maere S, De Bodt S, Raes J, Casneuf T, Van Montagu M, Kuiper M, Van de Peer Y (2005). Modeling gene and genome duplications in eukaryotes. Proc Natl Acad Sci.

[37] Wang G, Yin H, Qiao X, Tan X, Gu C, Wang B, Cheng R, Wang Y, Zhang S (2016). F-box genes: genome-wide expansion, evolution and their contribution to pollen growth in pear (*Pyrus bretschneideri*). Plant Sci.

[38] Starr TK, Jameson SC, Hogquist KA (2003). Positive and negative selection of T cells. Annu Rev Immunol.

[39] Yang ZH (2007). PAML 4: phylogenetic analysis by maximum likelihood. Mol Biol Evol.

[40] Saha J, Chatterjee C, Sengupta A, Gupta K, Gupta B (2014). Genome-wide analysis and evolutionary study of sucrose non-fermenting 1-related protein kinase 2 (SnRK2) gene family members in Arabidopsis and Oryza. Comput Biol Chem.

[41] Fujita Y, Nakashima K, Yoshida T, Katagiri T, Kidokoro S, Kanamori N, Umezawa T, Fujita M, Maruyama K, Ishiyama K (2009). Three SnRK2 protein kinases are the Main positive regulators of Abscisic acid signaling in response to water stress in Arabidopsis. Plant Cell Physiol.

[42] Fujii H, Verslues PE, Zhu JK (2007). Identification of two protein kinases required for abscisic acid regulation of seed germination, root growth, and gene expression in Arabidopsis. Plant Cell.

[43] Mustilli AC, Merlot S, Vavasseur A, Fenzi F, Giraudat J (2002). Arabidopsis OST1 protein kinase mediates the regulation of stomatal aperture by abscisic acid and acts upstream of reactive oxygen species production. Plant Cell.

[44] Boudsocq M, Barbier-Brygoo H, Lauriere C (2004). Identification of nine sucrose nonfermenting 1-related protein kinases 2 activated by hyperosmotic and saline stresses in Arabidopsis thaliana. J Biol Chem.

[45] Mizoguchi M (2010). Umezawa, Taishi, Nakashima, Kazuo, Kidokoro, Satoshi, Takasaki, Hironori: two closely related subclass II SnRK2 protein kinases cooperatively regulate drought-inducible gene expression. Plant Cell Physiol.

[46] Bai J, Mao J, Yang H, Khan A, Fan A, Liu S, Zhang J, Wang D, Gao H, Zhang J (2017). Sucrose non-ferment 1 related protein kinase 2 (SnRK2) genes could mediate the stress responses in potato (*Solanum tuberosum* L.). BMC Genet.

[47] Wang Y, Li X, Lin Y, Wang Y, Wang K, Sun C, Lu T, Zhang M (2017). Structural variation, functional differentiation, and activity correlation of the cytochrome P450 gene superfamily revealed in ginseng. Plant Genome.

[48] Liu Z, Ge X, Yang Z, Zhang C, Zhao G, Chen E. Genome-wide identification and characterization of SnRK2 gene family in cotton ( *Gossypium hirsutum* L.). BMC Genet. 2017;18(1). 10.1186/s12863-017-0517-3 PMC5469022 28606097

[49] Eddy SR (2011). Accelerated profile HMM searches. PLoS Comput Biol.

[50] Tamura K, Stecher G, Peterson D, Filipski A, Kumar S (2013). MEGA6: molecular evolutionary genetics analysis version 6.0. Mol Biol Evol.

[51] Bailey TL, Williams N, Misleh C, Li WW (2006). MEME: discovering and analyzing DNA and protein sequence motifs. Nucleic Acids Res.

[52] Lee TH, Tang HB, Wang XY, Paterson AH (2013). PGDD: a database of gene and genome duplication in plants. Nucleic Acids Res.

[53] Tang HB, Wang XY, Bowers JE, Ming R, Alam M, Paterson AH (2008). Unraveling ancient hexaploidy through multiply-aligned angiosperm gene maps. Genome Res.

[54] Krzywinski M, Schein J, Birol I, Connors J, Gascoyne R, Horsman D, Jones SJ, Marra MA (2009). Circos: an information aesthetic for comparative genomics. Genome Res.

