# Supplementary figures and images for: Genome-wide survey of sucrose non-fermenting 1-related protein kinase 2 in Rosaceae and expression analysis of PbrSnRK2 in response to ABA stress
Source: BMC Genomics. 2020 Nov 10;21:781. doi: 10.1186/s12864-020-07201-w (PMC7653828; doi:10.1186/s12864-020-07201-w)

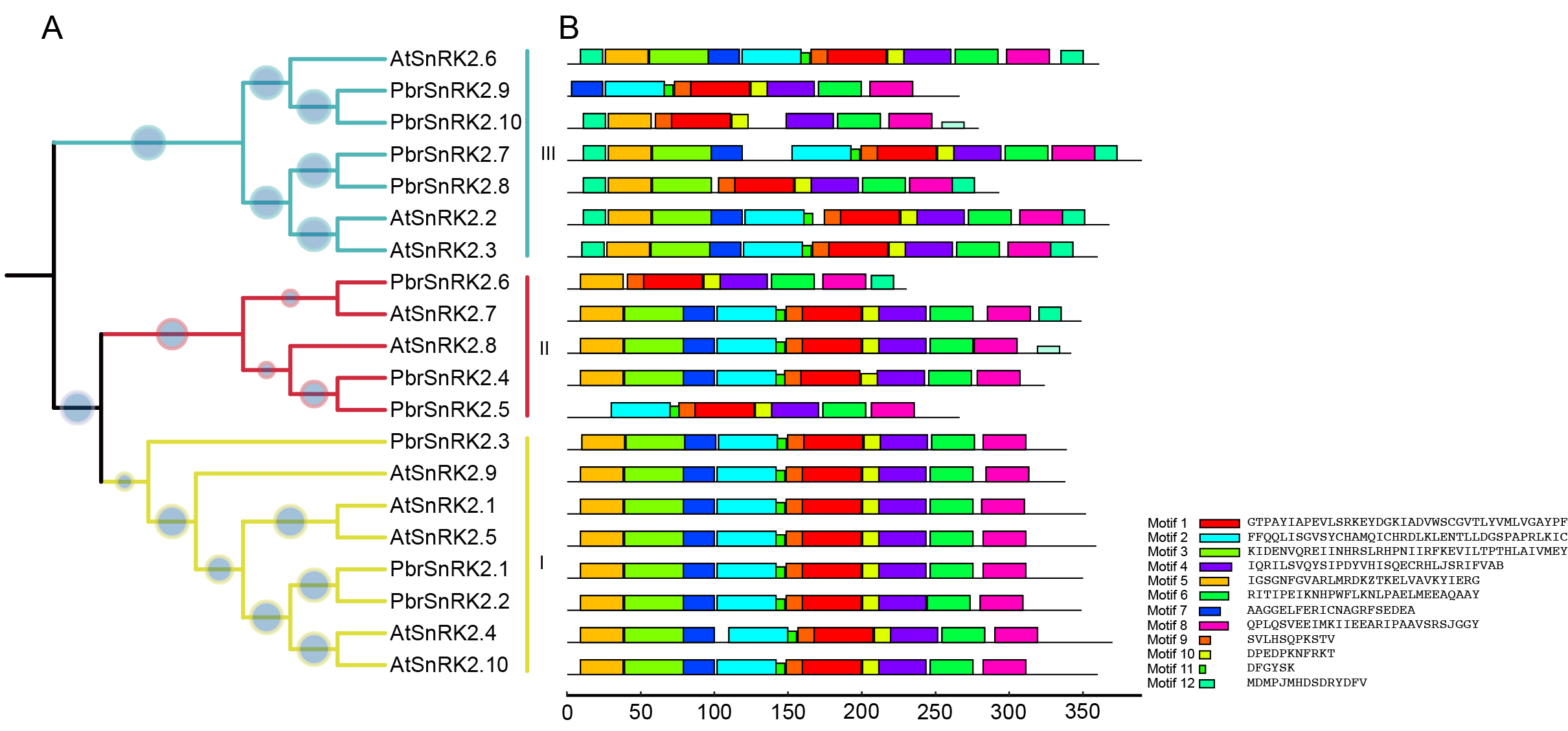

Supplement: Supplementary file 1 — Additional file 1: Figure S1. Phylogenetic relationship of conserved motif of SnRK2 genes from pear and Arabidopsis thaliana. A.A phylogenetic tree constructed with CluxtalX2.0 using the full-length amino acid sequences of SnRK2 genes from pear and Arabidopsis. Bootstrap analysis was performed using 1000 replicates. The species in which SnRK2 proteins were functionally characterized are displayed as icons, and the different colors in the branches represent the different subfamilies. B. MEME tools were used to identify conserved motifs of SnRK2 proteins, different colors represent different motifs. The scale bar indicates 50 aa. [file 12864_2020_7201_MOESM1_ESM.tif]

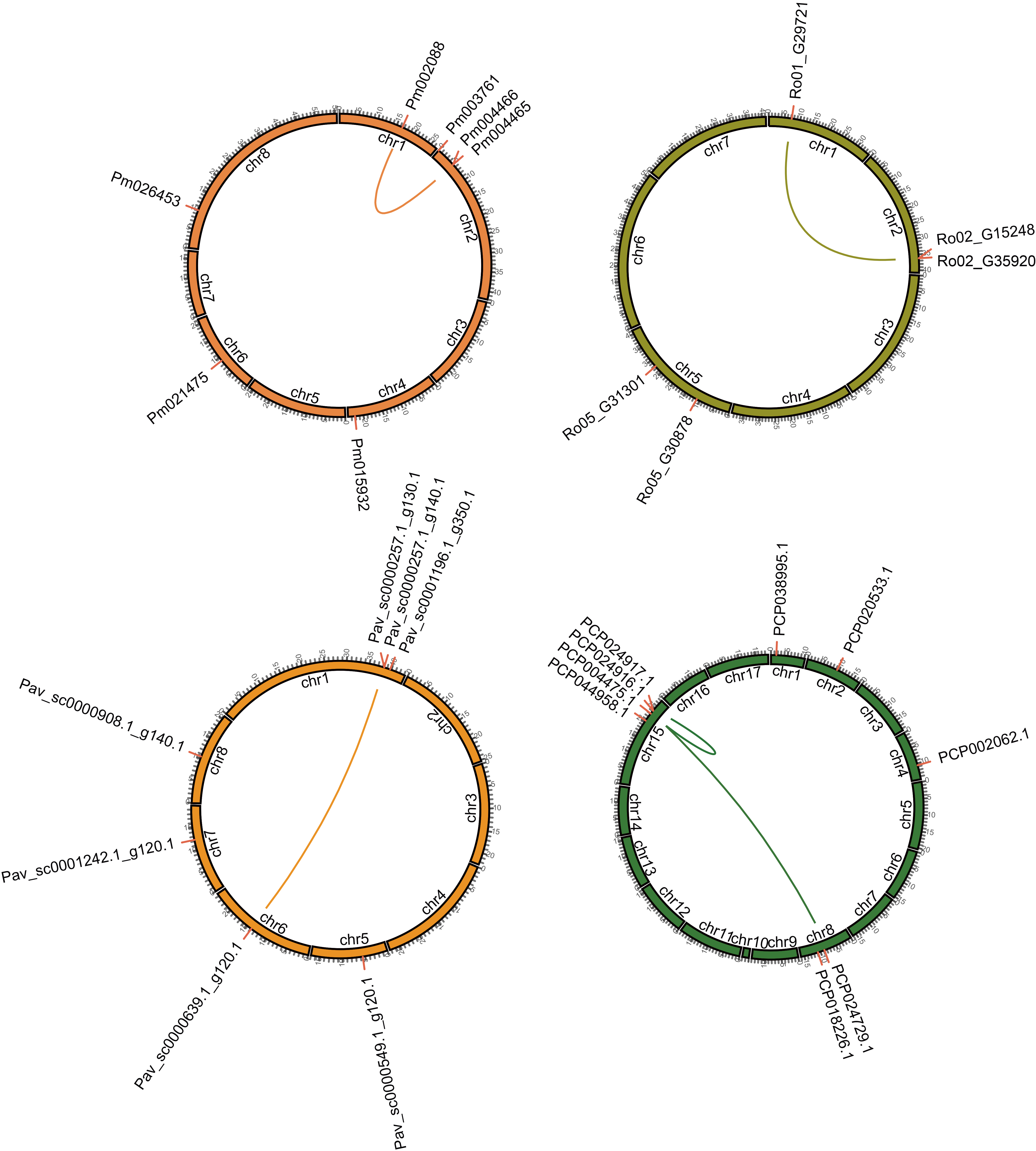

Supplement: Supplementary file 2 — Additional file 2: Figure S2. Chromosomal localization and synteny of SnRK2 genes in Rosaceae genomes. SnRK2 genes in Chinese plum, black raspberry, cherry, and European pear were mapped onto different chromosomes. Chromosome number is indicated on the inner side in the inner circle corresponding to different SnRK2 genes. Gene pairs with a syntenic relationship are joined by a line. [file 12864_2020_7201_MOESM2_ESM.tif]

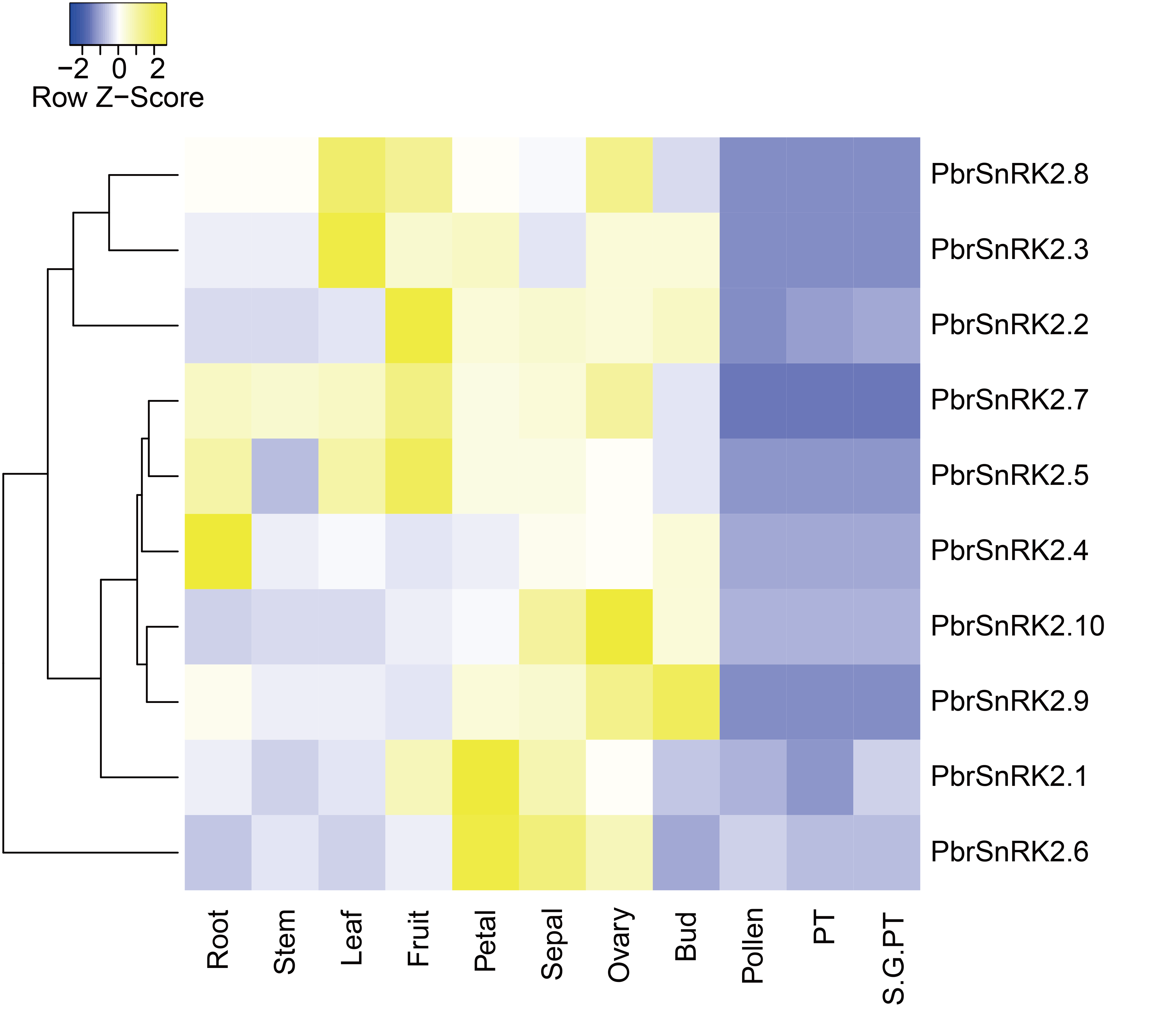

Supplement: Supplementary file 3 — Additional file 3: Figure S3. Analysis of the expression levels of PbrSnRK2 in different pear tissues. A heat map depicting the overall trend of the differential expression profiles of PbrSnRK2 genes in different pear tissues was constructed using MeV. The rows in the heat map represent genes and columns represent tissues. The colors of heat map cells indicate the scaled expression levels of genes across different tissues. The color gradient from blue to yellow corresponds to low-to-high transcript levels. [file 12864_2020_7201_MOESM3_ESM.tif]
